# Morphological multiparameter filtration and persistent homology in mitochondrial image analysis

**DOI:** 10.1371/journal.pone.0310157

**Published:** 2024-09-20

**Authors:** Yu-Min Chung, Chuan-Shen Hu, Emily Sun, Henry C. Tseng

**Affiliations:** 1 Eli Lilly and Company, Indianapolis, IN, United States of America; 2 School of Physical and Mathematical Sciences, Nanyang Technological University, Singapore, Singapore; 3 Columbia Ophthalmology, Columbia University Irving Medical Center, New York, NY, United States of America; 4 Duke Eye Center, Department of Ophthalmology, Duke University Medical Center, Durham, NC, United States of America; Telethon Institute of Genetics and Medicine, ITALY

## Abstract

The complexity of branching and curvilinear morphology of a complete mitochondrial network within each cell is challenging to analyze and quantify. To address this challenge, we developed an image analysis technique using persistent homology with a multiparameter filtration framework, combining image processing techniques in mathematical morphology. We show that such filtrations contain both topological and geometric information about complex cellular organelle structures, which allows a software program to extract meaningful features. Using this information, we also develop a connectivity index that describes the morphology of the branching patterns. As proof of concept, we utilize this approach to study how mitochondrial networks are altered by genetic changes in the Optineurin gene. Mutations in the autophagy gene Optineurin (OPTN) are associated with primary open-angle glaucoma (POAG), amyotrophic lateral sclerosis (ALS), and Paget’s disease of the bone, but the pathophysiological mechanism is unclear. We utilized the proposed mathematical morphology-based multiparameter filtration and persistent homology approach to analyze and quantitatively compare how changes in the OPTN gene alter mitochondrial structures from their normal interconnected, tubular morphology into scattered, fragmented pieces.

## Introduction

Recent advances in cellular biology have revealed that the majority of intracellular organelles are not distinct units with regular geometric shapes. They appear as complex, irregular three-dimensional (3-D) structures with complex branching patterns or “holes” within structures devoid of organelle material. For example, mitochondria are more than individual ovoid-like structures but are found within an extensive network of curvilinear and branching tubular structures. Smaller mitochondria pieces are continuously separated from the main mitochondrial network in a process called fission when senescent or damaged in order to be degraded. On the other hand, two distinct mitochondria can combine and merge their inner and outer mitochondrial membranes in a process called fusion. When performing quantitative analysis of the shape and size of these organelles, it is difficult to study morphological changes in the overall geometry of the irregular mitochondrial network between cells. This challenge is exacerbated by differences in the shape of cells, which in turn influences organelle geometry. Current analytic methodologies are limited in their ability to take into account the overall irregular shapes for quantitation and to compare differences between cells. Therefore, the purpose of our work is to describe our novel mathematical representation of complex biological organelle shapes using persistent homology based on the multiparameter filtration [1, 2] and demonstrate the utility of our analysis methodology in analyzing morphological differences in intracellular organelles such as mitochondria.

In order to study cells and quantify organelle size and density at the histological level, cellular tissue or a monolayer of cells is often immunostained with a fluorescent dye, mounted on a slide, and imaged with a fluorescence microscope. The advent of the confocal microscope allows cells to be imaged in a thin optical “section” through various parts of the cells. Multiple optical histological sections can be obtained in exquisite detail. Since fluorescence intensity is proportional to the amount of organelle protein or material stained, the fluorescence signal is quantitative and can be analyzed by software for comparison between different samples.

Current software analysis of fluorescence staining of organelles is imprecise due to major limitations. First, to analyze fluorescence signals resulting from stained organelles, current approaches involve a “thresholding” step in which the user or the software determines the fluorescence signal intensity level that separates background noise from actual biologically relevant data. The software then uses the threshold level to calculate the total fluorescence signal and intensity for quantitation. This thresholding process is often performed manually by the researcher, and so can be quite arbitrary, subjective, and highly variable. Different threshold settings result in variability in the quantitation and subsequent comparative image analysis. This problem is exacerbated by high background staining or low fluorescence signal intensity. Secondly, it is important for the software to be able to determine cellular boundaries. Often, to allow relative comparison of organelle abundance among cells of different sizes, the fluorescence intensity of the organelle would need to be normalized by dividing by the cellular area. This process often requires human users to manually “draw” these cellular boundaries for the software, which introduces another subjective variability. Lastly, due to inherent variation in immunostaining experiments, one set of samples may be stained more intensely than other sets of experiments performed on a different day. What may appear to be noise in one set of experiments might appear as an actual fluorescence signal in another experiment. This often exacerbates the other two limitations mentioned above.

To address these limitations, we propose an approach to mathematically model a fluorescently stained organelle using both persistent homology (PH) and a multiparameter filtration (MF) method, aiming to reduce experimental and data analysis variability. PH, MF, and the induced multiparameter persistent homology (MPH) belong to the burgeoning field of Topological Data Analysis (TDA) (see, for instance, [2]). Recently, the PH framework has demonstrated its potential in microscopy image analysis, including applications in pathological bone [3], cancer-immune microenvironment [4], and the deep learning framework in medical image segmentation [5, 6]. On the other hand, the concept of MPH, representing a more general version of PH, has been well-established [7, 8], with studies applying MPH in diverse areas, such as biomolecular data [9–11] and biomedical/biological image analysis [12]. Notably, research incorporating MPH into the analysis of microscopy images remains limited and rare. In this work, we propose a hybrid framework that considers PH features on MF, namely, the MF-PH framework, incorporating operations in mathematical morphology to encode local geometric and topological information within digital mitochondria images. Compared to conventional PH based on sub-level set filtrations, this approach offers more detailed and subtle features for exploring the structures of mitochondria networks. Additionally, the proposed MF-PH framework provides a concrete example of MF and its topological characteristics on image data and a foundation for further research on MPH in the TDA community.

This mathematical model is independent of cellular shape and size. Thus, it is capable of representing complex, irregular geometric shapes found in intracellular organelles. Moreover, this approach does not require thresholding of fluorescence signals and thus has a high robustness to noise/signal intensity ratio. As such, it is less affected by high noise, low fluorescence signals, or variability in fluorescence staining between different sets of experiments. Finally, we created specific biological indices that can be used to compare differences in size and morphological complexity of organelles between different cell types, genetic backgrounds, or experimental conditions. Our analytic tool has potential applications in research that study genetic, metabolic, pharmacological, age-related, and functional changes of complex intracellular organelles in disease research and drug development.

As proof of concept, we utilize this approach to study how mitochondrial networks are altered by genetic changes. Mutations in autophagy gene Optineurin (OPTN) are associated with primary open-angle glaucoma (POAG), amyotrophic lateral sclerosis (ALS), Paget’s disease of the bone, but the pathophysiological mechanism is unclear [13–16]. Recent studies have shown that OPTN may play an important role in regulating mitochondrial networks and as part of the mitophagy pathway [17–19]. To study how OPTN affects the mitochondrial structure, we studied cells with wildtype OPTN (WT) and ubiquitous genetic knockout of the mouse OPTN gene (KO). We hypothesized that loss of normal OPTN function disrupts mitochondrial morphology and utilized our MF-PH analytic approach to analyze genetic disruptions of the mitochondrial network—from its normal interconnected, tubular morphology into scattered, fragmented pieces. This was performed utilizing confocal microscopy images of primary cells derived from transgenic mice with genetic knockout of OPTN that we present.

## Materials

### Cell cultures

Primary mouse embryonic fibroblasts (MEFs) were purified from wild-type (WT), optineurin (OPTN), and OPTN knock-out (KO) transgenic mice previously generated in our laboratory [20, 21]. MEF cells from WT-OPTN and KO-OPTN mice were plated on sterile glass coverslips in 24 well plates. They were cultured at 37°C in a humidified 5% CO_2_ incubator in media consisting of DMEM that was supplemented with 10% FBS, and 10% penicillin-streptomycin and GlutaMAX (all culture media reagents were from Gibco/ThermoFisher). The cells were passaged between 75-85% confluence to maintain the cell line. Cultures were allowed to sit overnight after plating before any manipulation of the cultural environment was performed. Cells were fixed in 4% paraformaldehyde.

### Immunofluorescence staining

Following fixation, cells were washed with PBS, and incubated with a primary antibody of Tom20 (Santa Cruz Biotechnology) for mitochondria in 5% donkey serum and 5% Triton X-100 in PBS overnight. After several PBS washes, appropriate secondary antibodies conjugated to Alexa Fluor-488 (1:500) (Invitrogen) were added to the coverslips for 1h before mounting on coverslips with DAPI Vectashield mounting medium (Vector Labs).

### Confocal fluorescence microscopy

**Fig 1:**
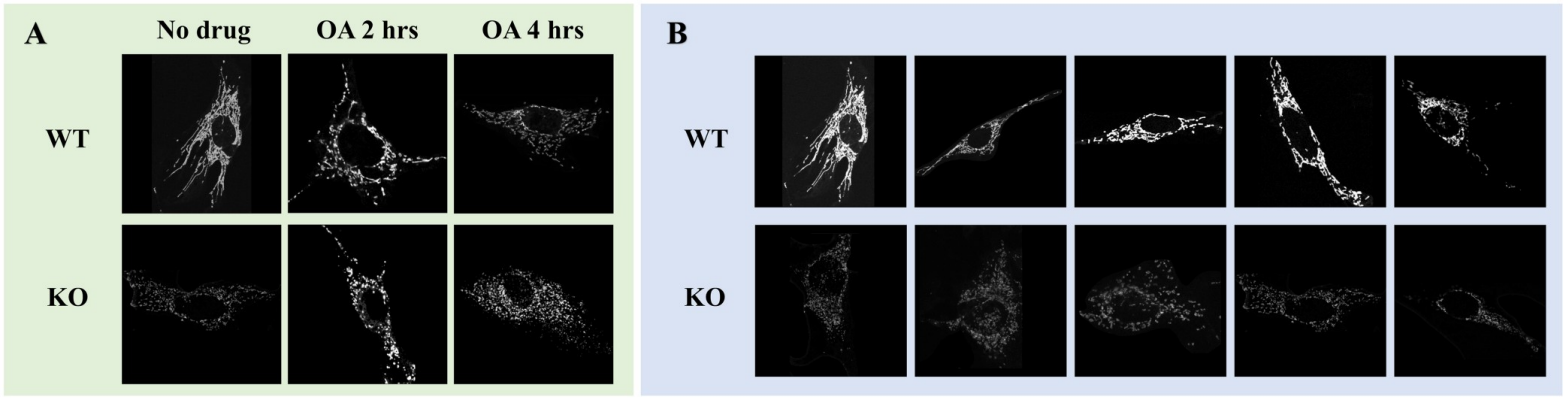
Microscopy images of mitochondrial cells. Panel A: Microscopy images of mitochondrial cells in classes WT and KO, along with images of cells treated with oligomycin and antimycin A (OA). Panel B: confocal microscopy images that will be studied in this work, where the first and second rows illustrate images of WT and KO types, respectively. For the presentation, images are padded with a black background to ensure proper alignment. The original images are included in the released materials for this work. For more details, refer to the data and code availability sections.

In the experiment of the work, two inhibitors for the mitochondrial cell are used, oligomycin and antimycin A, which impair mitochondrial respiration to induce mitochondrial damage and mitophagy. Especially, treatment with oligomycin and antimycin A for longer amounts of time should result in greater mitochondrial damage, subsequently leading to more fragmented and punctate mitochondrial networks. In this work, we consider two classes: WT and KO. Each class contains five images. In addition, we consider each class with oligomycin and antimycin A for 2 hours and 4 hours, respectively. The total images we have are 30 (5 × 3 × 2) images. Fig 1 demonstrates samples of confocal images. All cells were imaged with the same microscopy settings. However, fluorescence signals in KO cells appear lighter due to fragmentation of the mitochondrial network and decreased abundance of mitochondria proteins, particularly the Tom20 protein that was used as a marker for immunostaining. Mitochondrial network fragmentation is due to the genetic knockout of the optineurin gene.

Cells were imaged with a Nikon C2 Plus confocal microscope using a 63x NA oil immersion objective. A 488 nm laser was used to excite AlexaFluor 488. Slides were imaged at a resolution of 2048 pixels by 2048 pixels with the pinhole set to 1.2 AU. An exposure time of 1.2 *μs* per pixel was used, and the average of two frame acquisitions was used as the raw microscope image. Regions of interest were selected and a series of z-sections were taken from the top to the cell bottom with intervals between sections set to 2 *μm*.

### Ethical approval

All mouse experiments were performed at Duke University after having received approval from the Institutional Animal Care & Use Committee (IACUC) and were conducted according to Association for Research in Vision and Ophthalmology guidelines.

## Methods

The goal of this work is to use multiparameter filtration-based one-persistent homology to analyze confocal images of mitochondrial networks. This section has two main objectives: first, to review the essential mathematical details needed to introduce the proposed methods, and second, to present the mathematical notations that will be used throughout the article. This section begins with a review of fundamental topics related to digital images, including the use of binary and grayscale images and the mathematical modeling of complex shapes observed in mitochondrial morphology. It then covers the concept of Betti numbers and how they are assigned to a given binary image. Following this, we introduce and apply the concept of filtration, and discuss persistent homology. An overview of notations is provided in Table 1. For more mathematical background, we refer readers to these publications [1, 7, 22–24].

**Table 1:** Notations used in this article and their descriptions.

Notation	Description
⊆	Subset relation.
*P*	Set of pixels.
*f*	A binary image as a function from *P* to {0, 1}.
*K*(*f*)	{(*x*, *y*) ∈ *P* ∣ *f*(*x*, *y*) = 0}, the set of the black pixels of *f*.
*g*	A grayscale image as a function *g* : *P* → {0, 1, 2, 3, ⋯, 255}.
*g*_*t*_	The thresholded (binary) image of *g* at threshold value *t*.
*S*_*i*_	An (*i* + 1) × (*i* + 1) square as structural element.
$O_{S_{i}}\left(f\right)$	Opening operation on *f* with the structural element *S*_*i*_.
*β*_0_(*K*(*f*))	0-th Betti number of *K*(*f*). The number of isolated black regions in *f*.
*β*_1_(*K*(*f*))	1-st Betti number of *K*(*f*). The number of white regions enclosed by black regions in *f*.
${\left\{X_{i}\right\}}_{i=0}^{n}$	Filtration: a collection of sets *X*_*i*_ with the property *X*_1_ ⊆ *X*_2_ ⊆ ⋯ ⊆ *X*_*n*_.
${\left\{K\left(g_{t}\right)\right\}}_{t=0}^{255}$	Sublevel set filtration of a grayscale image *g*.
${\left\{K\left(O_{S_{i}}\left(f\right)\right)\right\}}_{i=0}^{20}$	Opening filtration of a binary image *f*.
$P({\left\{X_{i}\right\}}_{i=0}^{n})$	The persistence diagram of a given filtration ${\left\{X_{i}\right\}}_{i=0}^{n}$.
*X*_(*t*,*i*)_	$K(O_{S_{i}}\left(g_{t}\right))$ . The black pixel set of thresholded image *g*_*t*_ with opening $O_{S_{i}}$.
${\left\{X_{\left(t,i\right)}\right\}}_{i=0}^{n}$	Bifiltration: a collection of sets that satisfy subset relations, i.e., $X_{\left(t_{1},i_{1}\right)}\subseteq X_{\left(t_{2},i_{2}\right)}$ for any *t*_1_ ≤ *t*_2_ and *i*_1_ ≤ *i*_2_.
*X*_(*s*,*i*,*k*)_	$K(O_{T_{k}}\left(O_{S_{i}}\left(g_{s}\right)\right))$ . The black pixel set of thresholded image *g*_*s*_ with opening $O_{S_{i}}$ and $O_{T_{k}}$.
{*X*_(*s*,*i*,*k*)_}	A 3-variable indexed multiparameter filtration of sets with the relations, i.e., *X*_(*s*,*i*,*k*)_ ⊆ *X*_(*t*,*j*,*l*)_ for any *s* ≤ *t*, *i* ≤ *j*, and *k* ≤ *l*.
$P_{1}^{H,i}$	$P_{1}\left({\left\{X_{\left(t,i\right)}\right\}}_{t=0}^{255}\right)$ . Persistence diagram along the horizontal direction.
$P_{1}^{V,i}$	$P_{1}\left({\left\{X_{\left(t,i\right)}\right\}}_{i=0}^{20}\right)$ . Persistence diagram along the vertical direction.
*D*_*t*_	$\left\{d\;|\;\left(0,d\right)\in P_{1}^{V,t}\right\}$ . Multiset of size information.

### Digital images

**Fig 2:**
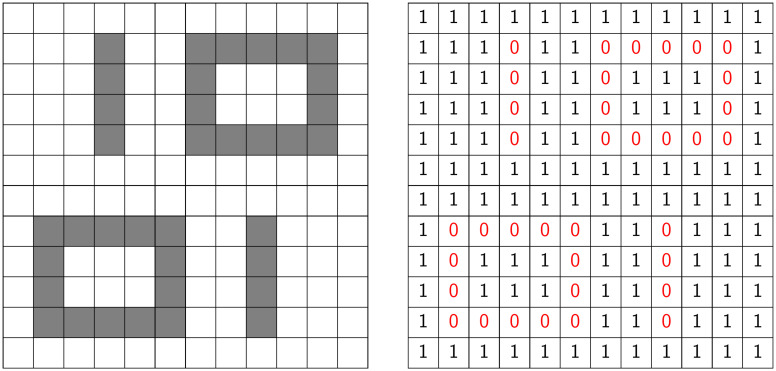
Illustration of a binary image and its function representation. The collection of black and white pixels for this binary image *f* is the 12-by-12 grid denoted by *P* = {(*x*, *y*) | *x*, *y* = 1, 2, 3, ⋯, 12}. Values 0 and 1 represent the black and white color, respectively. The set of black pixels is denoted by *K*(*f*).

Digital images consist of discrete grid points called pixels. Each pixel is a square and has a coordinate. The set of 2-dimensional integer lattice $Z^{2}$ is a common mathematical object to model the coordinate of pixels. Since digital images have fixed width and height, we use *P* which is a subset of $Z^{2}$ ($P\subseteq Z^{2}$) to denote the collection of pixels. For instance, Fig 2 shows a 12-by-12 binary image, and thus, *P* is the set of those 12-by-12 grids (pixels).

A binary image is then a function $f:P\subseteq Z^{2}\to \left\{0,\;1\right\}$, where 0 (1) usually represents the black (white, respectively) color. In this work, our focus will be the shapes formed by black pixels, i.e., the collection of pixels whose value is 0, formally denoted by *K*(*f*) ≔ {(*x*, *y*) ∈ *P* | *f*(*x*, *y*) = 0}. Interpreting the black pixels as black cubes within the $R^{2}$ plane, *K*(*f*) can be recognized as a subspace of $R^{2}$, commonly referred to as a cubical complex [25]. Reviewing Fig 2 once more, the left side illustrates the visualization of a binary image, while the right side depicts the corresponding function values of the binary image *f*. Moreover, in this example, *K*(*f*) represents the black regions (shape of “1” and “0”).

**Fig 3:**
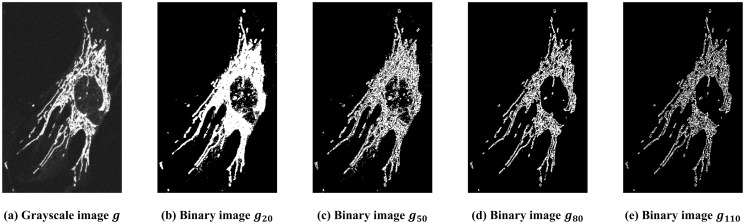
Illustration of the thresholding process. (a) A grayscale image of a WT cell. (b) ∼ (e) Binary images of *g* at threshold 20, 50, 80, and 110, respectively. Black sets grow as threshold increases, i.e., *K*(*g*_20_) ⊆ *K*(*g*_50_) ⊆ *K*(*g*_80_) ⊆ *K*(*g*_110_).

Grayscale images are the extension of binary images. Confocal images of the mitochondrial network in this study are grayscale images. Formally, an 8-bit grayscale image is a function $g:P\subseteq Z^{2}\to \left\{0,1,2,\cdots ,255\right\}$. The main difference between a grayscale and a binary image is that the pixel values of a grayscale image range from 0 to 255 while those of a binary image range are either 0 or 1. Figs 1 and 3(a) show examples of grayscale images. Pixel value 0 represents black while 255 represents white; values in between 1 and 255 represent the gray color gradient from black to white.

Converting a grayscale image to a binary image has been described in computer vision [26, 27]. In this work, we consider the most common way of doing this: a global thresholding method, which means that pixel values below a certain threshold are set to 0 and 1 otherwise. In other words, pixel values below a certain threshold are set to black and white otherwise. Mathematically, it can be described as follows: Given a grayscale image *g* and a threshold value *t*, the thresholded binary image of *g* at *t* is defined as

$$\begin{array}{c} g_{t}\left(x,y\right)=\{\begin{array}{c} 0,\;\text{if}\;g(x,y)\leq t\\ 1,\;\text{Otherwise}\; \end{array}. \end{array}$$ (1)

Consequently, given an 8-bit grayscale image *g*, global thresholding produces a total of 256 binary images: *g*_0_, *g*_1_, ⋯, *g*_255_. Fig 3(b)–3(e) show the binary image at different threshold values. Selecting one threshold is a challenging task and is often subjective (see e.g. [28–30]. On the other hand, our proposed method takes all threshold values into consideration.

At this point, we have seen binary, grayscale images, and their interaction via the global thresholding method in (1). Digital image processing concerns the manipulation of binary or grayscale images. Among many tools in the field, we consider a classic topic Mathematical Morphology that offers a wide range of methods to analyze images [31–38].

Methods in mathematical morphology are often called morphological operations. The building block of morphological operations is the structuring element. Because morphological operations are concerned with interactions among pixels, in particular local information around given pixels, structuring elements are the medium to acquire such information. We consider the *opening* operation associated with the square structuring element in this work. We denote it by $O_{S_{i}}\left(f\right)$, where *f* is a given binary image, and *S*_*i*_ is the square structuring element of size (*i* + 1) × (*i* + 1). Specifically, *S*_*i*_ can be depicted below

$$\begin{array}{c} S_{0}=\begin{array}{c} \circ \end{array}\;\;,\;\;S_{1}=\begin{array}{cc} • & •\\ \circ & • \end{array}\;\;,\;\;S_{2}=\begin{array}{ccc} • & • & •\\ • & \circ & •\\ • & • & • \end{array}\;\;,\;\;S_{3}=\begin{array}{cccc} • & • & • & •\\ • & • & • & •\\ • & \circ & • & •\\ • & • & • & • \end{array}\;\;,\;\cdots ,\;\; \end{array}$$ (2)

where the white bullet ∘ denotes the original point (0, 0) in $Z^{2}$.

**Fig 4:**
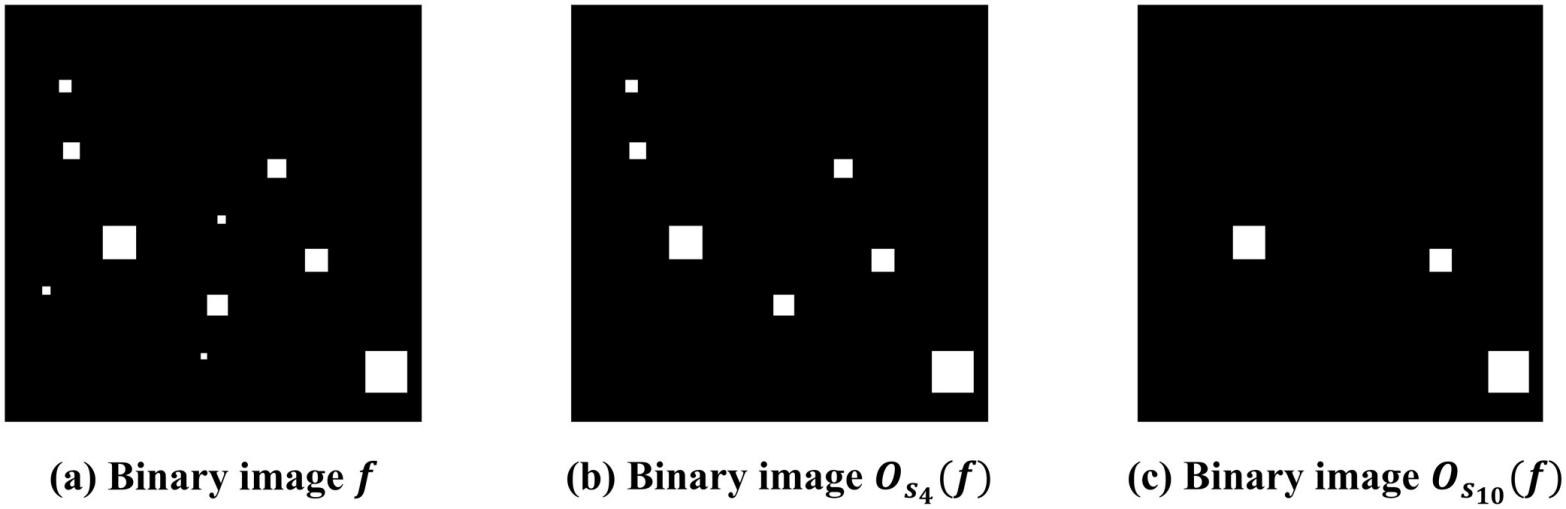
Illustration of opening operations with square structural elements. The structural elements used in the illustration are defined in (2). (a) The original binary image *f*. (b)-(c) The processed binary image $O_{S_{4}}\left(f\right)$ ($O_{S_{10}}\left(f\right)$) by opening operation with structural element *S*_4_ (*S*_10_, respectively). (a), (b), and (c) form a filtration. Black sets grow as the structuring element grows, i.e., $K\left(f\right)\subseteq K\left(O_{S_{4}}\left(f\right)\right)\subseteq K\left(O_{S_{10}}\left(f\right)\right)$.

One may think of the opening operation as a transformation that takes the given binary image and outputs the processed binary image. In other words, given a binary image *f* and a structural element *S*, applying the opening operation *O*_*S*_ to *f* results in another binary image, denoted by *O*_*S*_(*f*). Note that when *S* = *S*_0_, the opening operation *O*_*S*_ is identity, i.e., $O_{S_{0}}\left(f\right)=f$, and that $O_{S_{i}}\left(f\right)$ is also a binary image. Its formal mathematical definition can be found in these publications [1, 31–33]. Opening operation is commonly used to reduce noises in the binary image. By its design, an opening operation removes isolated white regions whose size is less than the structural element *S*. For instance, Fig 4(a) shows a binary image with various isolated white regions. We see that in Fig 4(b), those white regions whose size are less than (4 + 1) × (4 + 1) are removed by the opening operation $O_{S_{4}}\left(f\right)$. Similarly, in Fig 4(c), those white regions whose size are less than (10 + 1) × (10 + 1) are removed by $O_{S_{10}}\left(f\right)$. Selecting the “correct” structural element in practice is often a challenging task performed manually by the researcher and is subjective. Instead, our proposed method utilizes information from all parameters.

### Betti numbers

The *Betti numbers* constitute a classic subject in the field of algebraic topology in mathematics [39–42]. They play a crucial role in quantifying objects based on their connectivity, as well as in characterizing loop and void structures. They have been used in digital image processing [43–47]. Recently, the Betti numbers of one-parameter persistent homology were discovered to exhibit capabilities in identifying and segmenting tasks within microscope image analysis [48]. In this subsection, we will explain the meaning of Betti numbers for binary images.

**Fig 5:**
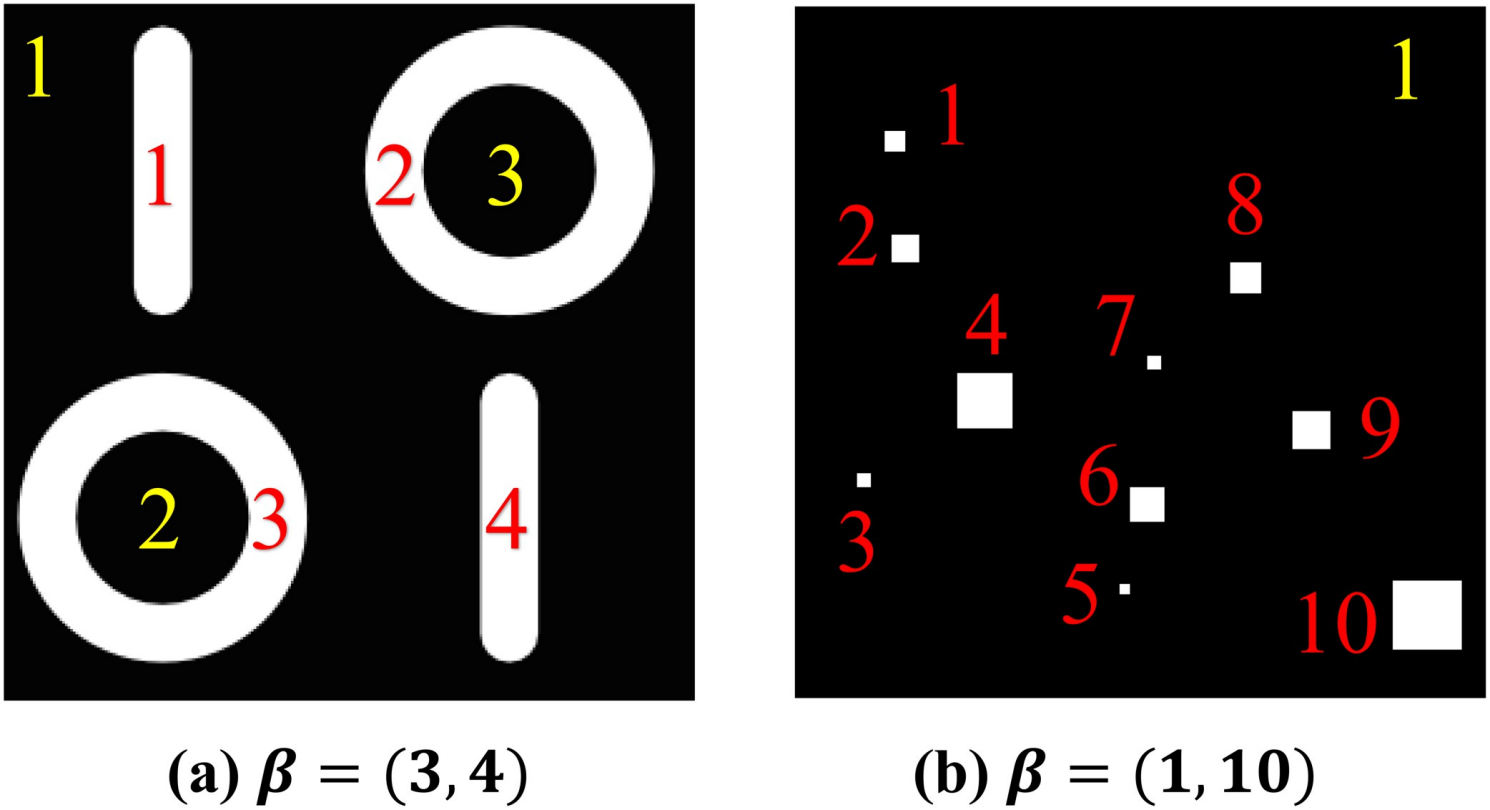
Sample binary images and Betti numbers of black regions. *β*_0_ counts the number of isolated black regions (counted by yellow numbers in (a) and (b), respectively), and *β*_1_ counts the number of white regions that are completely enclosed by the black ones (counted by red numbers in (a) and (b), respectively).

Let *f* be a binary image. We consider its black region, *K*(*f*). Betti numbers of *K*(*f*), denoted by *β*(*K*(*f*)), is a pair of numbers (*β*_0_, *β*_1_) where *β*_0_ (*β*_1_) is called 0-th (1-st) level Betti numbers. *β*_0_ counts the number of isolated black regions, and *β*_1_ counts the number of white regions that are completely enclosed by the black ones. Take Fig 5(a) as an example. Since there are three disjoint clusters of black pixels (background, two small black circles inside the white ones), *β*_0_ = 3; since there are four clusters of white pixels that are completely enclosed by the black ones, *β*_1_ = 4. Similarly, in Fig 5(b), we observe there are ten white regions that are completely enclosed by the black pixels, and thus, *β* = (1, 10).

Betti numbers are known to be invariant under translation, rotation, shrinking, or enlarging (cf. [39], Ch. 20, p. 128). For instance, as shown in Fig 5(a), a white vertical bar and a white circle are different shapes and sizes, but they all count equally towards *β*_1_. To echo its property, consider the binary image in Fig 5(b). There are various sizes of the white regions from a size of 3 × 3 to 20 × 20, but they all count equally towards the *β*_1_.

**Fig 6:**
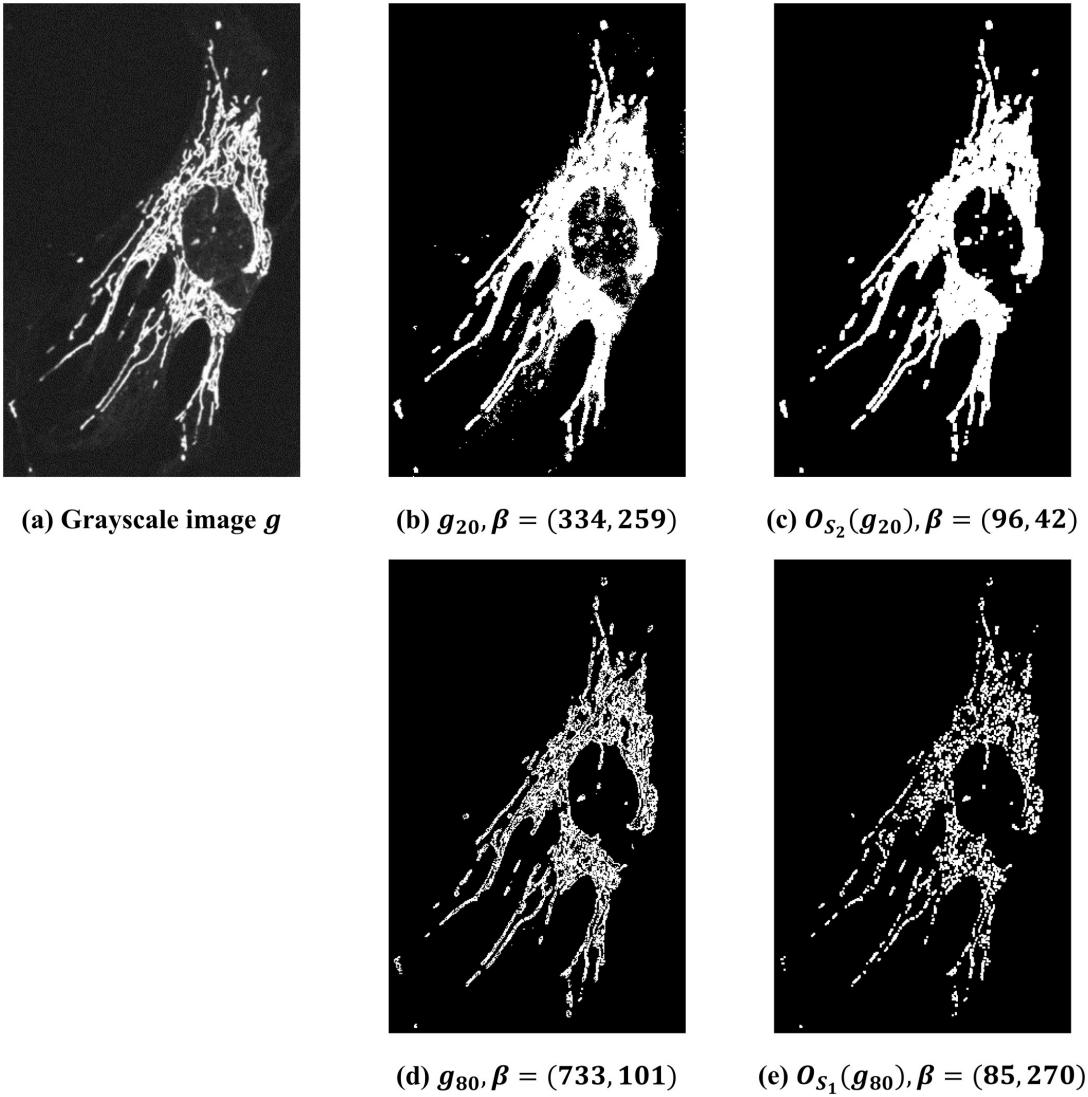
Illustration of thresholding, opening operation, and Betti numbers. (a) The original grayscale image of a WT cell. (b) Thresholded binary image of *g* at 20. Betti number of this binary image is *β* = (334, 259). (c) Opening operation with respect to *S*_2_ on *g*_20_. Betti number of this binary image is *β* = (96, 42). (d) Thresholded binary image of *g* at 80. Betti number of this binary image is *β* = (733, 101). (e) Opening operation with respect to *S*_1_ on *g*_80_. Betti number of this binary image is *β* = (85, 270).

Lastly, we demonstrate the interplay among global thresholding, opening operations, and Betti numbers. Consider Fig 6, where *g* is a grayscale image of a WT cell. First, let us consider what happens to Betti numbers when we use current analysis techniques employing manual thresholding. Fig 6(b) shows the thresholded binary image at 20 and its Betti number is (334, 259). We apply the opening operation with respect to *S*_2_ on the binary image *g*_20_ to obtain Fig 6(c), and its Betti number is (96, 42). We observe that the binary image $O_{S_{2}}\left(g_{20}\right)$ visually is cleaner than *g*_20_ and their Betti numbers change drastically. As a comparison, Fig 6(d) shows the thresholded binary image at 80. It seems that *g*_80_ reveals a finer structure of the cells than *g*_20_ does. We apply the opening operation with respect to *S*_1_ on the binary image *g*_80_ to obtain Fig 6(e). At this point, we observe that there are two thresholding parameters in this process, and choosing different pairs of parameters results in different images and Betti numbers. Thus, setting the manual threshold is often subjective, and differences in thresholding will change the analysis outcome. To address this challenge, our proposed MF approach, on the other hand, takes all possible thresholding parameters into consideration so that it offers a global view of the images.

### Filtration

At the end of the previous subsection, we observe that different thresholds or different structural elements would result in different binary images. To address this, we will utilize the *Persistent Homology* (PH), a recently developed tool in the field of topological data analysis [7, 49–54]. We first introduce the concept of filtration, which is essential for understanding persistent homology.

Filtration is a sequence of sets where sets satisfy subset relations. Mathematically, let *X*_*i*_ be some set with index *i*. In our context, we think of *X*_*i*_ as the black set of the binary image. Consider a family of sets ${\left\{X_{i}\right\}}_{i=0}^{n}$ and we say that ${\left\{X_{i}\right\}}_{i=0}^{n}$ is a filtration if

$$\begin{array}{c} X_{0}\subseteq X_{1}\subseteq X_{2}\subseteq \cdots \subseteq X_{n}. \end{array}$$ (3)

The definition means that the set *X*_*i*_ grows as the index *i* increases. In our context, since we consider the black sets of binary images, filtration of black sets means the binary image becomes darker as the index increases. There are two filtrations in this work: sublevel set filtration and opening filtration.

The first filtration is related to the global thresholding method in (1). Given an 8-bit grayscale image *g*, recall from (1), *g*_*t*_ is the thresholded binary image at threshold *t*. We consider the set of black pixels of *g*_*t*_, i.e., *K*(*g*_*t*_). The *sublevel set filtration* (cf. [53], Sec. VI.1, p. 125) is the following nested subset relations:

$$\begin{array}{c} K\left(g_{0}\right)\subseteq K\left(g_{1}\right)\subseteq K\left(g_{2}\right)\subseteq \cdots \subseteq K\left(g_{255}\right). \end{array}$$ (4)

The idea is that as the threshold value increases, the corresponding binary image becomes darker (in the extreme case, when the threshold value is 255, then the binary image is completely black). This can be seen in Fig 3(b)–3(e) where the set of black pixels grows as the threshold increases. Given any grayscale image *g*, the sublevel set filtration of *g* is diagram (4) and is denoted by ${\left\{K\left(g_{t}\right)\right\}}_{t=0}^{255}$.

The second filtration is related to the opening operation. As discussed in the previous subsection, opening filtration with the structural element *S*_*i*_ can be thought of as filtering those isolated white regions whose size is less than (*i* + 1) × (*i* + 1). Thus, as *S*_*i*_ becomes larger, more white regions will be removed by the opening operation; for instance, it can be seen in Fig 4. We now consider a family of opening operations $O_{S_{i}}$ for *i* = 0, 1, 2, ⋯, 20. As shown in [1, 55], this family of operations possesses the subset relations called the *opening filtration*:

$$\begin{array}{c} K(O_{S_{0}}\left(f\right))\subseteq K\left(O_{S_{1}}\left(f\right)\right)\subseteq K\left(O_{S_{2}}\left(f\right)\right)\subseteq \cdots \subseteq K\left(O_{S_{20}}\left(f\right)\right), \end{array}$$ (5)

where *f* is a given binary image. The opening filtration of *f* is diagram (5) and is denoted by ${\left\{K\left(O_{S_{i}}\left(f\right)\right)\right\}}_{i=0}^{20}$.

### Persistent homology

Here, we introduce the idea of persistent homology [51, 53, 54] and its representation: persistence diagrams. At this point, we have seen Betti numbers of the binary images. Persistent homology can be thought of as the generalization of the Betti numbers. Formal mathematical developments of persistent homology can be found in [51, 53, 54]. In this subsection, we will use an example to demonstrate persistent homology.

Filtration of sets is the essential assumption for persistent homology. Given a filtration ${\left\{X_{i}\right\}}_{i=0}^{n}$ persistent homology not only computes Betti numbers of each set but also tracks the changes of Betti numbers. To see persistent homology in action, we demonstrate it via a concrete example of a grayscale image below.

gx,y=1321102132. (6)

**Fig 7:**
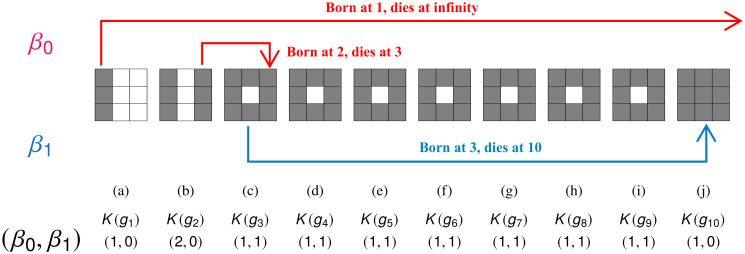
Illustration of persistent homology. Grayscale image *g* is defined in (6). (a)-(j) Sublevel sets (or binary images) of *g* at threshold 1, 2, 3, ⋯, 10, respectively. (Top panel *β*_0_) Changes of *β*_0_ features. (Bottom panel *β*_1_) Changes of *β*_1_ features. The sets of black pixels in the binary images, denoted as *K*(*g*_1_), …, *K*(*g*_10_), form a filtration of cubical complexes. Persistence diagrams of *g* are $P_{0}=\left\{\left(1,\infty \right),\left(2,3\right)\right\}$ and $P_{1}=\left\{\left(3,10\right)\right\}$.

The corresponding threshold decomposition, i.e., all possible binary images of *g*, are shown in Fig 7.

To determine persistent homology, we calculate Betti numbers at each thresholded binary image *K*(*g*_*t*_) and track changes in Betti numbers because of the filtration relation. Note that the Betti numbers for each *K*(*g*_*t*_) can be found in Fig 7 and also that there are three changes of Betti numbers, i.e., (1) from *K*(*g*_1_) to *K*(*g*_2_) where Betti numbers change from (1, 0) to (2, 0), (2) from *K*(*g*_2_) to *K*(*g*_3_) where Betti numbers change from (2, 0) to (1, 1), and (3) from *K*(*g*_9_) to *K*(*g*_10_) where Betti numbers change from (1, 1) to (1, 0). Persistent homology keeps track of these changes in Betti numbers.

On the ground level, we will look into how pixels form components/holes, or fill up the grid. In Fig 7, since at *K*(*g*_1_) there is a newly formed black column, we say that “a *β*_0_ feature is born at *K*(*g*_1_)”. Similarly, at *K*(*g*_2_), there is another newly formed black column, and another *β*_0_ feature is born at *K*(*g*_2_). At *K*(*g*_3_), two changes occur. One is that the *β*_0_ feature that is born at *K*(*g*_2_) joins with the other *β*_0_ feature. Thus, we say that this generator dies at *K*(*g*_3_). For this generator, we record its birth and death time as a pair of numbers (2, 3), where the first (second) component is the birth (death) value. We also observe that at *K*(*g*_3_), *β*_1_ changes from 0 to 1, and thus, a *β*_1_ generator is born at *K*(*g*_3_). From *K*(*g*_4_) to *K*(*g*_9_), black sets do not change. Finally, at *K*(*g*_10_), the entire image becomes black and thus the Betti number is (1, 0). A *β*_1_ feature dies at *K*(*g*_10_), and its birth-death pair would be (3, 10). Note that the first *β*_0_ generator persists in this process, so by convention, we assign its death value by ∞ (cf. [53], Sec. VII.1, p. 152), its birth-death pair is (1, ∞). Collections of such birth-death pairs are called persistence diagrams with respect to the filtration. The persistence diagrams associated with the dimensions of connected components and loops are termed the persistence diagrams in dimensions 0 and 1, respectively. We use the following notation: $P_{0}({\left\{K\left(g_{t}\right)\right\}}_{t=1}^{10})=\left\{\left(1,\infty \right),\left(2,3\right)\right\}$ and $P_{1}({\left\{K\left(g_{t}\right)\right\}}_{t=1}^{10})=\left\{\left(3,10\right)\right\}$. To summarize this subsection, given a filtration of sets ${\left\{X_{i}\right\}}_{i=0}^{n}$, there is a corresponding persistence diagram denoted by $P({\left\{X_{i}\right\}}_{i=0}^{n})$. There are several sophisticated software packages to compute persistence diagrams for a given filtration (see a survey article [56] for a list of software). Among them, we use Perseus software package [57, 58] to compute persistence diagrams in this work.

### Multiparameter filtration construction

At this point, we have seen that given a filtration, one can study persistence diagrams. In particular, such filtration is with respect to one parameter, such as threshold values in sublevel set filtration in (3) or the sizes of structural elements in the opening filtration (5). It is also known as one-parameter filtration, and the corresponding persistence diagrams are called one-parameter persistence. Recently, there have been studies to generalize this concept to multiparameter filtration and multiparameter persistence [22–24, 59–61]. The proposed model will utilize two-parameter filtration or bifiltration. In what follows, we will introduce the proposed model. The core idea of the proposed model is to combine sublevel set filtration in (3) and opening filtration in (5).

To get started, we review the formal definition of a bifiltration (cf. [22], Sec. 2). Similar to one-parameter filtration discussed in (3), a bifiltration is a sequence of sets {*X*_(*i*,*j*)_} along with two parameters such that

$$\begin{array}{c} X_{\left(i_{1},j_{1}\right)}\subseteq X_{\left(i_{2},j_{2}\right)},\;\forall \;i_{1}\leq i_{2},\;j_{1}\leq j_{2}. \end{array}$$ (7)

We observe that instead of a scalar in (3), the index in (7) is now a pair of numbers. As a demonstration, consider the following bifiltration {*X*_(*i*,*j*)_} where *i* = 0, 1, 2, 3 and *j* = 0, 1, 2:

$$\begin{array}{ccccccc} X_{\left(0,0\right)} & \subseteq & X_{\left(1,0\right)} & \subseteq & X_{\left(2,0\right)} & \subseteq & X_{\left(3,0\right)}\\ |\;\cap & & |\;\cap & & |\;\cap & & |\;\cap \\ X_{\left(0,1\right)} & \subseteq & X_{\left(1,1\right)} & \subseteq & X_{\left(2,1\right)} & \subseteq & X_{\left(3,1\right)}\\ |\;\cap & & |\;\cap & & |\;\cap & & |\;\cap \\ X_{\left(0,2\right)} & \subseteq & X_{\left(1,2\right)} & \subseteq & X_{\left(2,2\right)} & \subseteq & X_{\left(3,2\right)} \end{array}.$$ (8)

In the example of diagram (8), for instance, *X*_(0,0)_ is a subset of *X*_(1,0)_ because 0 ≤ 1 and 0 ≤ 0. Similarly, *X*_(1,1)_ is a subset of *X*_(3,2)_ because 1 ≤ 3 and 1 ≤ 2. On the other hand, since 2 ≤ 3 but 1 ≰ 0, the relation *X*_(2,1)_ ⊆ *X*_(3,0)_ is not guaranteed. We may also observe that each row (and column) in (8) is a one-parameter filtration.

We are now ready to present our proposed model. Given a grayscale image *g*, the proposed model combines sublevel set filtration in (4) and opening filtration in (5) to form a bifiltration. The rigorous consideration and its mathematical proof can be found in [1]. Intuitively, one may start with the sublevel set filtration $K\left(g_{i_{1}}\right)\subseteq K\left(g_{i_{2}}\right)$ as long as *i*_1_ ≤ *i*_2_. Observe that since for fixed *i*, *g*_*i*_ is a binary image, one may apply opening operations to *g*_*i*_ to obtain the opening filtration $K\left(O_{S_{j_{1}}}\left(g_{i}\right)\right)\subseteq K\left(O_{S_{j_{2}}}\left(g_{i}\right)\right)$ for any *j*_1_ ≤ *j*_2_. Therefore, for each binary image *g*_*i*_, there is a corresponding opening filtration; moreover, each *g*_*i*_ satisfies the sublevel set filtration. Thus, we obtain the following bifiltration:

$$\begin{array}{ccccccc} K(g_{0}) & \subseteq & K(g_{1}) & \subseteq \cdots \subseteq & K(g_{254}) & \subseteq & K(g_{255})\\ |\;\cap & & |\;\cap & \vdots & |\;\cap & & |\;\cap \\ K(O_{S_{1}}\left(g_{0}\right)) & \subseteq & K(O_{S_{1}}\left(g_{1}\right)) & \subseteq \cdots \subseteq & K(O_{S_{1}}\left(g_{254}\right)) & \subseteq & K(O_{S_{1}}\left(g_{255}\right))\\ |\;\cap & & |\;\cap & \vdots & |\;\cap & & |\;\cap \\ K(O_{S_{2}}\left(g_{0}\right)) & \subseteq & K(O_{S_{2}}\left(g_{1}\right)) & \subseteq \cdots \subseteq & K(O_{S_{2}}\left(g_{254}\right)) & \subseteq & K(O_{S_{2}}\left(g_{255}\right))\\ |\;\cap & & |\;\cap & \vdots & |\;\cap & & |\;\cap \\ \vdots & & \vdots & \vdots & \vdots & & \vdots \\ |\;\cap & & |\;\cap & \vdots & |\;\cap & & |\;\cap \\ K(O_{S_{19}}\left(g_{0}\right)) & \subseteq & K(O_{S_{19}}\left(g_{1}\right)) & \subseteq \cdots \subseteq & K(O_{S_{19}}\left(g_{254}\right)) & \subseteq & K(O_{S_{19}}\left(g_{255}\right))\\ |\;\cap & & |\;\cap & \vdots & |\;\cap & & |\;\cap \\ K(O_{S_{20}}\left(g_{0}\right)) & \subseteq & K(O_{S_{20}}\left(g_{1}\right)) & \subseteq \cdots \subseteq & K(O_{S_{20}}\left(g_{254}\right)) & \subseteq & K(O_{S_{20}}\left(g_{255}\right)) \end{array}.$$ (9)

To simplify the notation, we denote $X_{\left(t,i\right)}:=K\left(O_{S_{i}}\left(g_{t}\right)\right)$. Then the above equation can be simplified as

$$\begin{array}{ccccccc} X_{\left(0,0\right)} & \subseteq & X_{\left(1,0\right)} & \subseteq \cdots \subseteq & X_{\left(254,0\right)} & \subseteq & X_{\left(255,0\right)}\\ |\;\cap & & |\;\cap & \vdots & |\;\cap & & |\;\cap \\ X_{\left(0,1\right)} & \subseteq & X_{\left(1,1\right)} & \subseteq \cdots \subseteq & X_{\left(254,1\right)} & \subseteq & X_{\left(255,1\right)}\\ |\;\cap & & |\;\cap & \vdots & |\;\cap & & |\;\cap \\ X_{\left(0,2\right)} & \subseteq & X_{\left(1,2\right)} & \subseteq \cdots \subseteq & X_{\left(254,2\right)} & \subseteq & X_{\left(255,2\right)}\\ |\;\cap & & |\;\cap & \vdots & |\;\cap & & |\;\cap \\ \vdots & & \vdots & \vdots & \vdots & & \vdots \\ |\;\cap & & |\;\cap & \vdots & |\;\cap & & |\;\cap \\ X_{\left(0,20\right)} & \subseteq & X_{\left(1,20\right)} & \subseteq \cdots \subseteq & X_{\left(254,20\right)} & \subseteq & X_{\left(255,20\right)} \end{array}.$$ (10)

**Fig 8:**
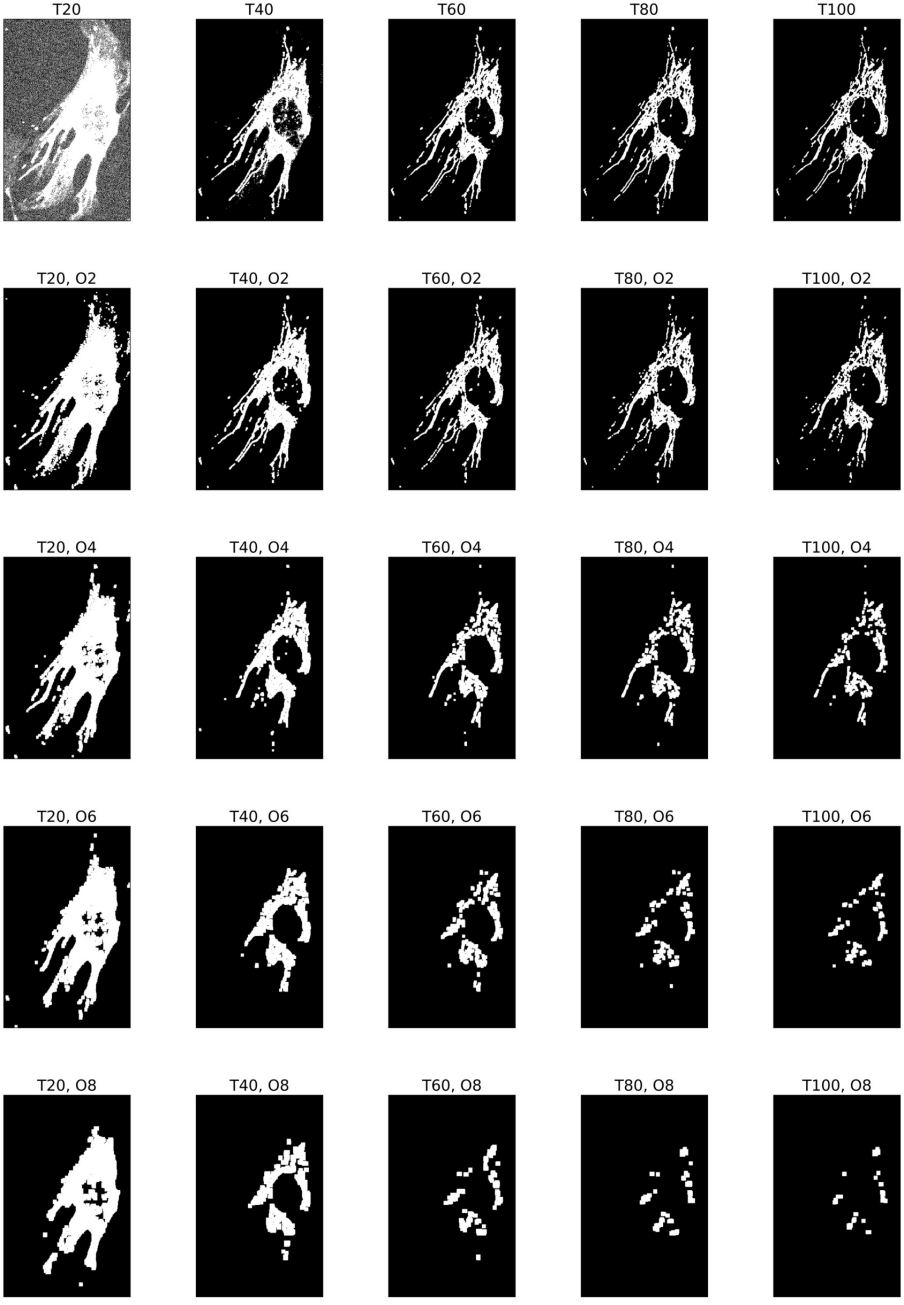
Illustration of a bifiltration of a WT image. The bifiltration is defined as in (10), where *T* = 20, 40, 60, 80, 100 means the threshold of image intensity, and *O* means the sizes of the opening operation corresponding structuring elements *S*_0_, *S*_2_, *S*_4_, *S*_6_, *S*_8_. The WT image is the first in the first row of Panel B in Fig 1.

**Fig 9:**
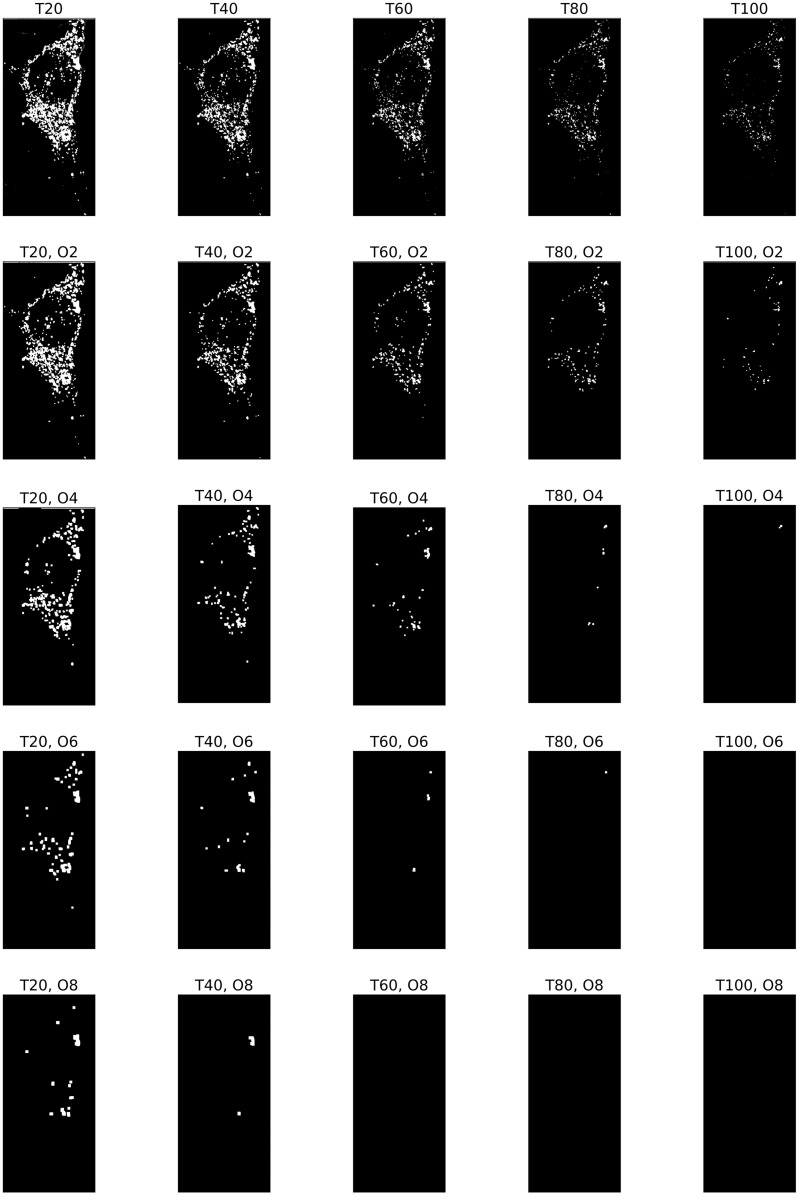
Illustration of a bifiltration of a KO image. The bifiltration is defined as in (10), where *T* = 20, 40, 60, 80, 100 means the threshold of image intensity, and *O* means the sizes of the opening operation corresponding structuring elements *S*_0_, *S*_2_, *S*_4_, *S*_6_, *S*_8_. The KO image is the first in the second row of Panel B in Fig 1.

The Eq (10) is our proposed persistent homology model based on multiparameter filtrations. Given a grayscale image *g*, we then consider the corresponding bifiltration. Figs 8 and 9 visualize the bifiltration in (10).

As discussed in the previous sections, given a filtration of sets, one may track changes in Betti numbers of these multisets in the form of persistence diagrams. Similarly, one would desire to possess analogous “persistence diagrams” for a multiparameter filtration. Unfortunately, the notion of persistence diagrams for a multiparameter filtration does not exist [8, 62], and hence, it is challenging to utilize its full power in practice. There have been works to devise ways for practical use. For example, the software RIVET [63] offers a framework for constructing a bifiltration for a given point cloud data and computes a 1-dimensional persistence diagram along any path in the bifiltration [12] built upon RIVET to develop a multiparameter landscape which can be seen as extracting information from the bifiltration. In our study, we aim to extract features from the bifiltration as well. The main difference is that our approach is specifically for image data (cubical complexes) as opposed to point cloud data (simplicial complexes). In addition, the ways of constructing bifiltration as we describe in this article are by morphological operations. In the next section, we will describe our proposed features from the proposed bifiltration. We summarize this section by stating the proposed pipeline:

$$\begin{array}{c} \text{Input}\;\text{a}\;\text{grayscale}\;\text{image}\;\to \text{Construct}\;\text{bifiltration}\;(10)\to \text{Extract}\;\text{features}. \end{array}$$ (11)

Beyond bifiltrations, the proposed MF construction based on morphological operations can be generalized to MF with more parameters. For instance, suppose *S*_1_ ⊆ *S*_2_ ⊆ ⋯ ⊆ *S*_*n*_ and *T*_1_ ⊆ *T*_2_ ⊆ ⋯ ⊆ *T*_*n*_ are two increasing sequences of structuring elements as required in [1, 55]. Then, the inclusion relations $K\left(O_{S_{i}}\left(f\right)\right)\subseteq K\left(O_{S_{j}}\left(f\right)\right)$ and $K\left(O_{T_{k}}\left(f\right)\right)\subseteq K\left(O_{T_{l}}\left(f\right)\right)$ are valid for every binary image *f* : *P* → {0, 1}. Consequently, every grayscale image *g* admits the bifiltration

$$\begin{array}{ccc} K(O_{S_{i}}\left(g_{s}\right)) & \subseteq & K(O_{S_{j}}\left(g_{s}\right))\\ |\;\cap & & |\;\cap \\ K(O_{S_{i}}\left(g_{t}\right)) & \subseteq & K(O_{S_{j}}\left(g_{t}\right)) \end{array}$$ (12)

whenever *i* ≤ *j* and *s* ≤ *t* with *i*, *j* ∈ {1, 2, …, *n*} and *s*, *t* ∈ {0, 1, …, 255}. By applying operations $O_{T_{k}}$ (*k* = 1, 2, …, *m*) on this filtration, the following inclusion relations

$$\begin{array}{ccc} K(O_{T_{k}}\left(O_{S_{i}}\left(g_{s}\right)\right)) & \subseteq & K(O_{T_{l}}\left(O_{S_{j}}\left(g_{s}\right)\right))\\ |\;\cap & & |\;\cap \\ K(O_{T_{k}}\left(O_{S_{i}}\left(g_{t}\right)\right)) & \subseteq & K(O_{T_{l}}\left(O_{S_{j}}\left(g_{t}\right)\right)) \end{array}$$ (13)

arise for *k* ≤ *l*, *i* ≤ *j*, *s* ≤ *t* with *k*, *l* ∈ {1, 2, …, *m*}, *i*, *j* ∈ {1, 2, …, *n*}, and *s*, *t* ∈ {0, 1, …, 255}. Similarly, by defining $X_{\left(s,i,k\right)}=K\left(O_{T_{k}}\left(O_{S_{i}}\left(g_{s}\right)\right)\right)$ over these indices, the family {*X*_(*s*,*i*,*k*)_} forms a MF indexed by three parameters, i.e.,

$$\begin{array}{c} X_{\left(s,i,k\right)}\subseteq X_{\left(t,j,l\right)},\;\forall \;s\leq t,\;i\leq j,\;k\leq l. \end{array}$$ (14)

Actually, by leveraging different morphological operations (e.g., erosion, dilation, closing, etc. [32]), a multifiltration of images in any number of parameters can be produced [1]. However, to demonstrate multifiltration in a more geometrically intuitive and interpretable way, we focus on investigating the bifiltration described in (10) and compute the persistent homology on it.

The extraction of the PH information from the proposed bifiltration proceeds as follows. Note that each horizontal relation in (10) is a sublevel set filtration; each vertical relation in (10) is an opening filtration. Each horizontal contains topological information: how the grayscale image is formed as the threshold increases. Each vertical contains geometric information: the size of holes at each binary image. It is also possible to follow a path that is neither horizontal nor vertical, which could be an interesting topic for future research. In this study, however, we will focus on vertical and horizontal paths. More precisely, we consider

$$\begin{array}{cc} & P_{1}^{H,i}≔P_{1}\left({\left\{X_{\left(t,i\right)}\right\}}_{t=0}^{255}\right); \end{array}$$ (15)

$$\begin{array}{cc} & P_{1}^{V,t}≔P_{1}\left({\left\{X_{\left(t,i\right)}\right\}}_{i=0}^{20}\right). \end{array}$$ (16)

$P_{1}^{H,i}$ is the 1-st persistence diagram of the horizontal path for fixed *i*; $P_{1}^{V,t}$ is the 1-st persistence diagram of the vertical path for fixed *t*.

## Proposed features

As shown in the previous section, diagram (10) offers rich information about both the geometric and topological properties of the underlying images. In this section, we will describe the features from the bifiltration in (10) and apply them to the mitochondrial images. Each horizontal or vertical filtration contains different information about mitochondria. There are three main features that we will use in this work: (A) normalized Betti numbers curve, (B) size distribution of the Betti numbers, and (C) the connectivity index that we present. We will describe details of each feature in this section. These three features are summarized in Table 2.

**Table 2:** Features extracted from the proposed bifiltration in this article and their descriptions.

Features	Formula
(A) Normalized Betti curve	$\rho_{1}^{H,i}\left(t\right)=\frac{\#\beta_{1}\left(X_{\left(t,i\right)}\right)}{\#P_{1}^{H,i}}$
(B) Size distribution of Betti number	$\phi \left(D_{t}\right)=\text{histogram}(\frac{D_{t}}{\#D_{t}})$
(C) Connectivity index	$C_{t}:=\frac{\sum_{\left(b,d\right)\in P_{1}^{V,t}\backslash D_{t}}\left(d-b\right)}{\sum_{\left(b,d\right)\in P_{1}^{V,t}}\left(d-b\right)}$

### Normalized Betti curve

Betti curves have been used in several studies and shown to be effective shape descriptors [64–67]. *Normalized Betti curves*, on the other hand, are more recently introduced in the work by [65]. The essential idea is to calculate *β*_1_ for each thresholded binary image at *t* and normalize it by the total number of points in a persistence diagram. Compared to the classic Betti number curve, [65] showed that it possesses desired theoretic properties over the classic one. We refer interested readers to [65] for more details. Building upon this work, we are using normalized Betti curves in the context of bifiltration for our MF-PH analysis technique.

To illustrate our approach, let the opening parameter *i* be fixed and consider the horizontal persistence diagram in (15), $P_{1}^{H,i}$. The normalized Betti curve is defined for the *i*-th row of (10) as

$$\begin{array}{c} \rho_{1}^{H,i}\left(t\right)=\frac{\#\beta_{1}\left(X_{\left(t,i\right)}\right)}{\#P_{1}^{H,i}},\;\;\text{for}\;t=0,1,\cdots ,255, \end{array}$$ (17)

where # denotes the number of elements in a set or a multiset. By its design, we observe that the values of $\rho_{1}^{H,i}$ are between 0 and 1. Therefore, for any different raw image, this feature is both scale and size-independent. No preprocessing on the raw images is required in order to compare these curves.

### Size distribution of Betti numbers

Betti numbers are invariant under scaling, so objects with different sizes would count equally toward Betti numbers [39–42]. Because of our proposed bifiltration (10), such geometric information may be captured from the vertical filtration. This idea is similar to the so-called granumonetry [32, 68–70] in material science.

Our rationale is that since mitochondrial networks in KO cells are fragmented, these are expected to be smaller in size. In contrast, WT cells have mitochondrial networks that are not as fragmented, so consist of mitochondria of larger sizes. To quantitatively compare differences in sizes, this information is represented by the vertical direction in (10). We designed this feature based on the size of Betti numbers, and thus we will call it the *size distribution of Betti numbers*. For each *t*, we consider the vertical direction in (10) and the corresponding persistence diagram in (16) $P_{1}^{V,t}$. As studied in [1], the multiset of death values

$$\begin{array}{c} D_{t}=\{d\;|\;\left(0,d\right)\in P_{1}^{V,t}\} \end{array}$$ (18)

indicates the sizes of 1-st dimensional holes in the binary image *X*_*t*,0_. Therefore, the size distribution of the Betti numbers along *t*-th column of diagram (10) is defined as

$$\begin{array}{c} \phi \left(D_{t}\right)=\text{histogram}(\frac{D_{t}}{\#D_{t}}). \end{array}$$ (19)

Since our opening filtration is from 1 to 20, the number of bins in *ϕ*(*D*_*t*_) is 20. For instance, at the 1-st bin of *ϕ*(*D*_*t*_), it represents the percentage of Betti numbers of *X*_*t*,0_ that are of size 1 × 1; at the 2-nd bin of *ϕ*(*D*_*t*_), it represents the percentage of Betti numbers of *X*_*t*,0_ that are of size 2 × 2; at the 20-th bin of *ϕ*(*D*_*t*_), it represents the percentage of Betti numbers of *X*_*t*,0_ that are of size 20 × 20.

### Connectivity index

Mitochondrial fragmentation and loss of connectivity are crucial elements in the analysis of how mitochondrial morphology is altered in response to genetic, biological, pharmacological, or environmental changes. Therefore, we propose a feature called *connectivity index* to assess the connectivity of a mitochondrial network. By identifying *D*_*t*_ as a sub-multiset of $P_{1}^{V,t}$ via the mapping *d* ↦ (0, *d*), we design our connectivity index as

$$\begin{array}{c} C_{t}≔\frac{\sum_{\left(b,d\right)\in P_{1}^{V,t}\backslash D_{t}}\left(d-b\right)}{\sum_{\left(b,d\right)\in P_{1}^{V,t}}\left(d-b\right)}. \end{array}$$ (20)

Note that by definition, *C*_*t*_ is calculated along the *t*-th column filtration in (10). Furthermore, the porous structures with persistence intervals of the form (*b*, ∞) are excluded from the connectivity index computation.

By considering the set size of *D*_*t*_ and the lifespans *d* − *b* of the persistence intervals, the proposed connectivity index offers a description of the fragmentation and loss of connectivity of a given image. By definition, the values of *C*_*t*_ range from 0 to 1 for any fixed *t*. If *C*_*t*_ is closer to 1, the input image is more connected; on the other hand, if *C*_*t*_ is closer to 0, the input image is more fragmented. As mentioned in (18), *D*_*t*_ indicates the sizes of Betti numbers in the binary image *X*_(*t*,0)_. If $D_{t}=P_{1}^{V,t}$, then the size of 1-dimensional holes (or porous structures) in the binary image *X*_*t*,0_ can be determined exactly. For otherwise, $P_{1}^{V,t}\backslash D_{t}\neq \emptyset$ means there are additional holes that are coming off the original holes. In other words, the more features in the multiset $P_{1}^{V,t}\backslash D_{t}$, the more connected the original binary image *X*_(*t*,0)_.

**Fig 10:**
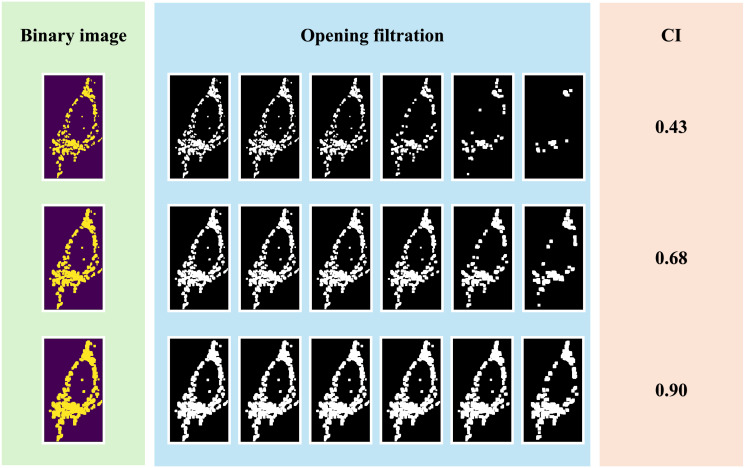
Illustration of opening filtrations and the corresponding connectivity indices. To illustrate different images’ fragmentation and connection structures, the binary images are derived from a mitochondrial image in class WT using morphological dilation and thresholding. CI refers to the connectivity index.

Fig 10 illustrates examples of three binary images to interpret the geometric insight of the connectivity index on digital images. The images located in the front rows have more sparse and fragmented (white) structures. As Betti-1 information, small porous structures as white pixels disappear as the black pixels increase in the opening filtration and correspond to smaller death values in the persistence diagram $P_{1}^{V,t}$. Furthermore, Betti-1 structures encoded as members in the set *D*_*t*_ have the birth value 0, representing the original structure in the image. For instance, for the first binary image in Fig 10, numerous porous structures exist in the original image, belonging to *D*_*t*_, leading to a smaller connectivity index (0.43). On the contrary, the third binary image is more robust and contains fewer singleton pore structures. Compared to the first and the second images, the cardinality of *D*_*t*_ is much smaller, leading to a higher connectivity index (0.9).

### Computational setup

We chose Perseus software to compute persistence diagrams because it offers executable files for all major operating systems (Windows, Linux, and Mac) and can be adapted to any coding environment [57]. Updates of the proposed framework in other packages (e.g., Gudhi [71]) can be found in our GitHub repository (see the “Code availability” section).

Using Perseus, computing a single persistent homology for the persistence diagram $P_{1}^{H,i}$ or $P_{1}^{V,i}$ for the proposed curve ($\rho_{1}^{H,0}\left(t\right)$, *ϕ*(*D*_*t*_), or *C*_*t*_) can be done in 10 to 20 seconds. The computation is based on a Windows 11 x64 environment with an AMD Ryzen 7 7840HS CPU and 16.0 GB of RAM.

## Results

This section presents the results for the proposed features on mitochondria images of types KO and WT: Normalized Betti Curve, Size Distribution of Betti Numbers, and Connectivity Index. The following paragraphs provide a detailed analysis of each feature.

### Normalized Betti curve

**Fig 11:**
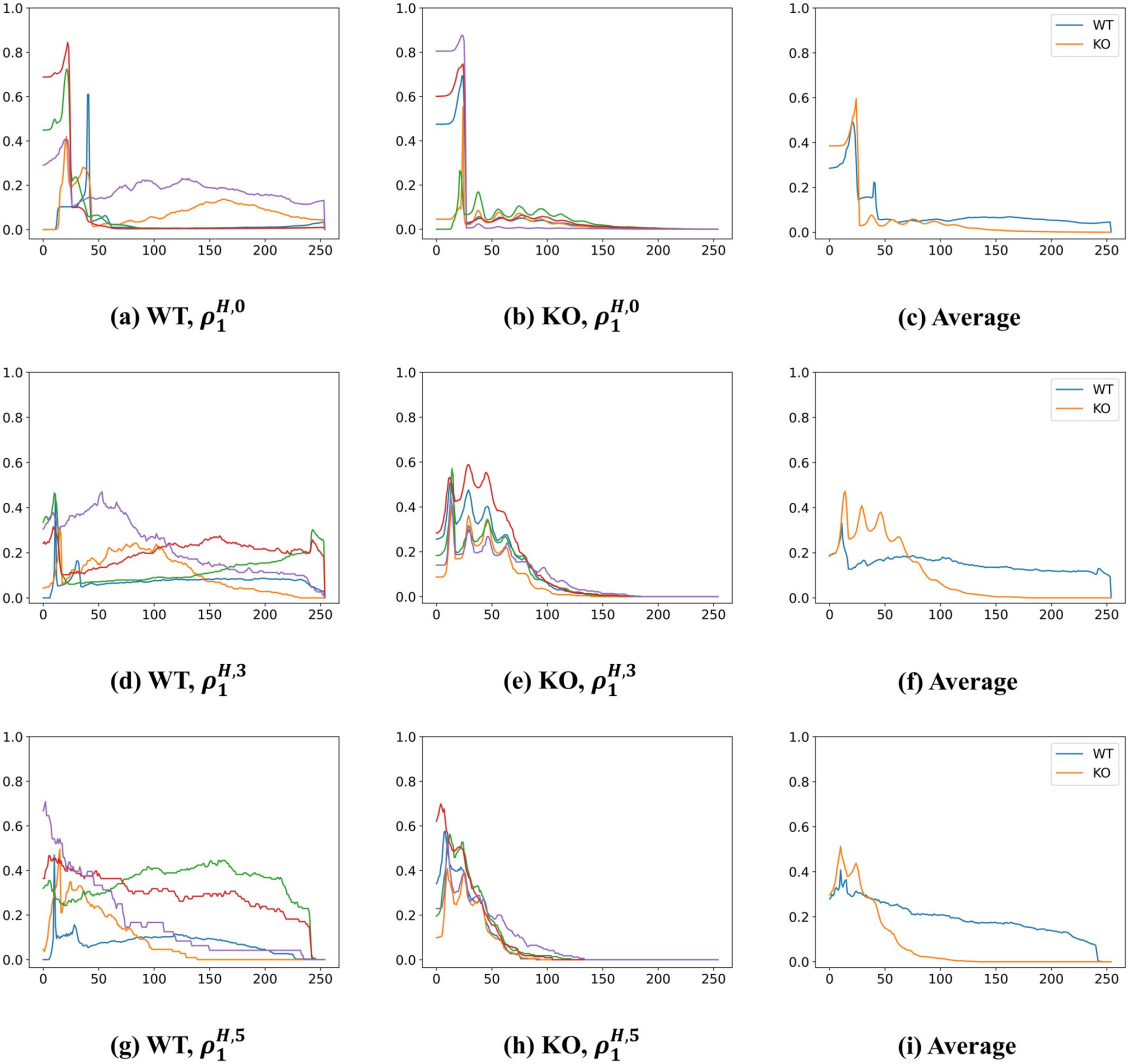
Comparison of normalized Betti curves for WT and KO images. The normalized Betti curves are defined as in (17), and the WT and KO images are shown in Fig 1. (a)-(b) normalized Betti curves for WT and KO respectively, $\rho_{1}^{H,0}$, along the horizontal (0-th row of diagram (10)) filtration ${\left\{X_{t,0}\right\}}_{t=0}^{255}$ where 0 indicates no opening operation is applied; (c) Average curves over those 5 images in (a) and (b); (d)-(e) normalized Betti curves for WT and KO respectively, $\rho_{1}^{H,3}$, along the horizontal (3-rd row of diagram (10)) filtration ${\left\{X_{t,3}\right\}}_{t=0}^{255}$ where 3 indicates opening operation with structural element *S*_3_ is applied; (f) Average curves over those 5 images in (d) and (e); (g)-(h) normalized Betti curves for WT and KO respectively, $\rho_{1}^{H,5}$, along the horizontal (5-th row of diagram (10)) filtration ${\left\{X_{t,5}\right\}}_{t=0}^{255}$ where 5 indicates opening operation with structural element *S*_5_ is applied; (i) Average curves over those 5 images in (g) and (h).

Fig 11 shows the normalized Betti curves in (17) for those images of WT and KO type cells as in Fig 1. Since there are 5 images (for 5 different cell samples) for each genotype, there are 5 curves in each Fig 11(a), 11(b), 11(d), 11(e), 11(g), and 11(h). We first consider the results of WT images. Fig 11(a), 11(d) and 11(g) show the normalized Betti curve at the 1-st, 4-th, and 6-th row of diagram (10): $\rho_{1}^{H,0}$, $\rho_{1}^{H,3}$, and $\rho_{1}^{H,5}$, respectively. Recall that each row of the diagram (10) indicates a different level of opening/smoothing where the 1-st row indicates no smoothing, the 4-th row indicates smoothing with $O_{S_{3}}$, and the 6-th row indicates smoothing with $O_{S_{5}}$. We observe the shapes of these curves are different among different WT cell samples. This suggests that mitochondrial morphologies in WT cells exhibit large variability.

Second, we consider the results of KO images. Fig 11(b), 11(e), and 11(h) show the normalized Betti curve at the 0-th, 3-rd, and 5-th row of diagram (10): $\rho_{1}^{H,0}$, $\rho_{1}^{H,3}$, and $\rho_{1}^{H,5}$, respectively. We observe that in (b), the shapes of these curves are similar among different sample images. Similar behaviors can also be observed in (e) and (h). The similarity in these curves suggests that all of these KO OPTN cells exhibit mitochondrial morphologies that are similar.

Lastly, we compare the curves between WT and KO types. To facilitate the comparison, we consider curves in Fig 11(c), 11(f) and 11(i) in which we generated curves representing averages for all the WT and KO cell samples. For instance, in (c), the blue curve represents the average curve over those 5 sample curves in Fig 11(a) while the red curve represents the average curve over those 5 curves in Fig 11(b). Similarly, the blue curves in (f) and (i) represent the average curve over those curves in (d) and (g), respectively. We observe that the difference in (c) is not obvious. However, in (f) and (i), the average curves for WT and KO are quite different. (c), (f), and (i) represent morphological comparisons across all gray-level thresholding. Therefore, our findings are consistent with what we see visually in these microscope images. This may suggest that normalized Betti curves are good descriptors for these two types of cells.

### Size distribution of Betti numbers

**Fig 12:**
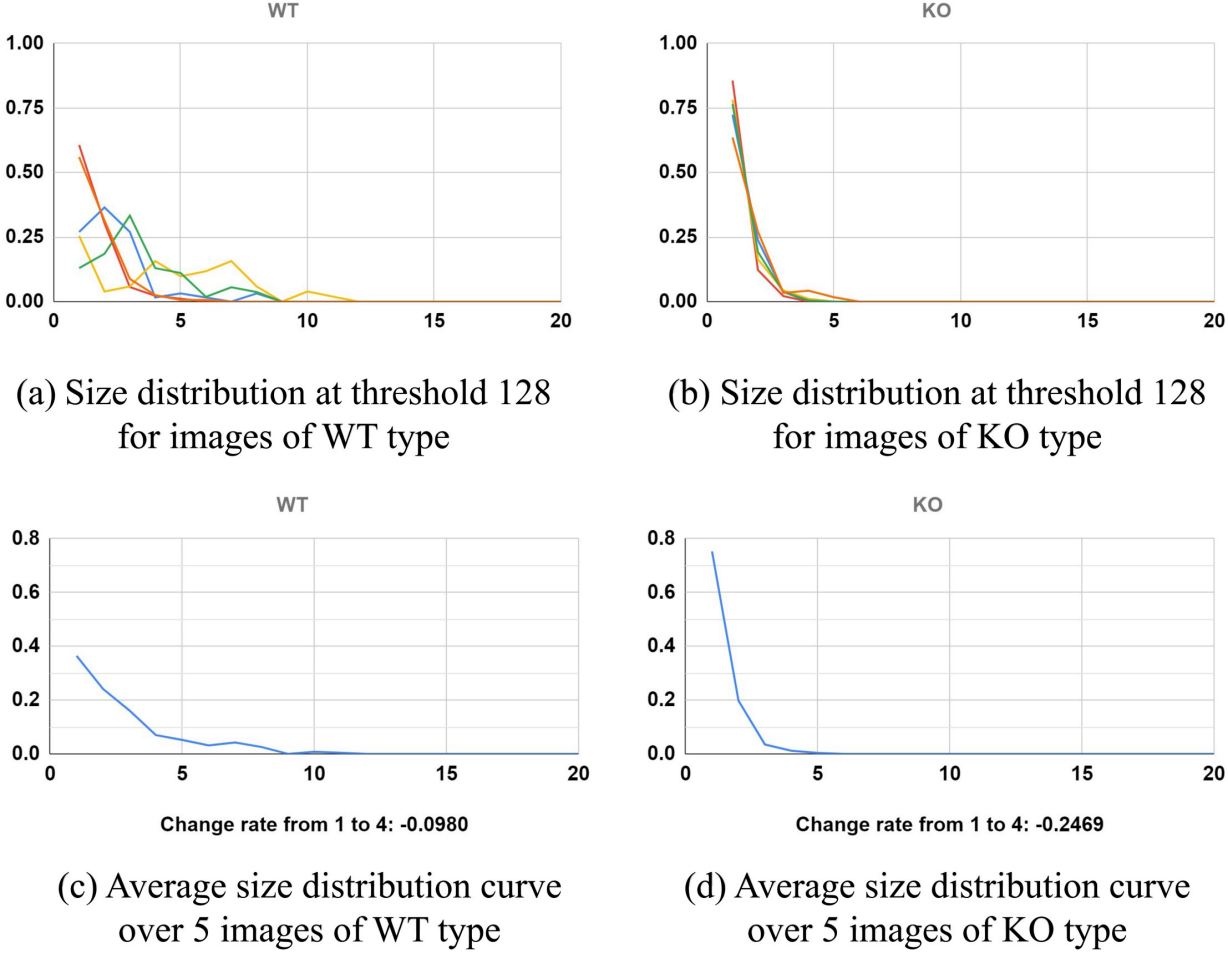
Comparison of size distributions of Betti numbers for WT and KO images. The size distributions of Betti numbers, *ϕ*(*D*_*t*_), are defined in (19), and the WT and KO images are shown in Fig 1. (a)-(b) *ϕ*(*D*_128_) of WT and KO, respectively, type. (c) Average over curves in (a). −0.0980 is the rate of change from size 1 to size 4. (d) Average over curves in (d). −0.2469 is the rate of change from size 1 to size 4.

We apply the size distribution of Betti numbers in (19) to quantify WT and KO. Fig 12(a) and 12(b) show the size distribution of Betti numbers along 128-th column of diagram (10), *ϕ*(*D*_128_), for WT and KO type, respectively. Each curve represents a single-cell sample; there are 5 different cells used per genotype. We observe that in Fig 12(a), among images in WT, the distributions seem to have more variability. For instance, the green curve in (a) shows that about 10% of Betti numbers are of size (1 + 1) × (1 + 1) (the first bin) and about 5% of Betti numbers are of size (7 + 1) × (7 + 1) (the 7-th bin); the red curve in (a) shows that about 50% of Betti numbers are of size (1 + 1) × (1 + 1) and none of the Betti numbers are beyond size (6 + 1) × (6 + 1). This behavior of high variability was also seen in the case of the normalized Betti curve.

On the other hand, in Fig 12(b), we observe that the distributions are very similar among the 5 sample images representing different cells for the KO genotype. For instance, curves in (b) show that more than 50% of Betti curves are of size (1 + 1) × (1 + 1), and almost none of them are of size (5 + 1) × (5 + 1) (zero after the 5-th bin). This reflects our expectation that mitochondrial networks in KO cells are more fragmented.

To further quantify the difference between WT and KO type, we calculated averages over curves in Fig 12(a) and 12(b), respectively. The average curve plots are shown in Fig 12(c) and 12(d), respectively. We observe that for the WT type, mitochondrial sizes of 1, 2, and 3 are about 40%, 20%, and 15%. On the other hand, for the KO type, they are about 70%, 15%, and 5%, which shows that a larger percentage of KO mitochondria are smaller and thus more fragmented than WT mitochondria. The results suggest that WT and KO have different size profiles. Furthermore, we also calculate the total rate of changes $\frac{\bar{\phi }\left(1\right)-\bar{\phi }\left(4\right)}{1-4}$ of the average curves $\bar{\phi }:\left\{1,...,20\right\}\to \left[0,1\right]$ from size 1 to size 4 which are −0.0980 and −0.2469 as displayed in Fig 12(c) and 12(d). The rate of change in mitochondria size in these curves is another way to measure differences in mitochondrial size distribution between the two cell types.

### Connectivity index

**Fig 13:**
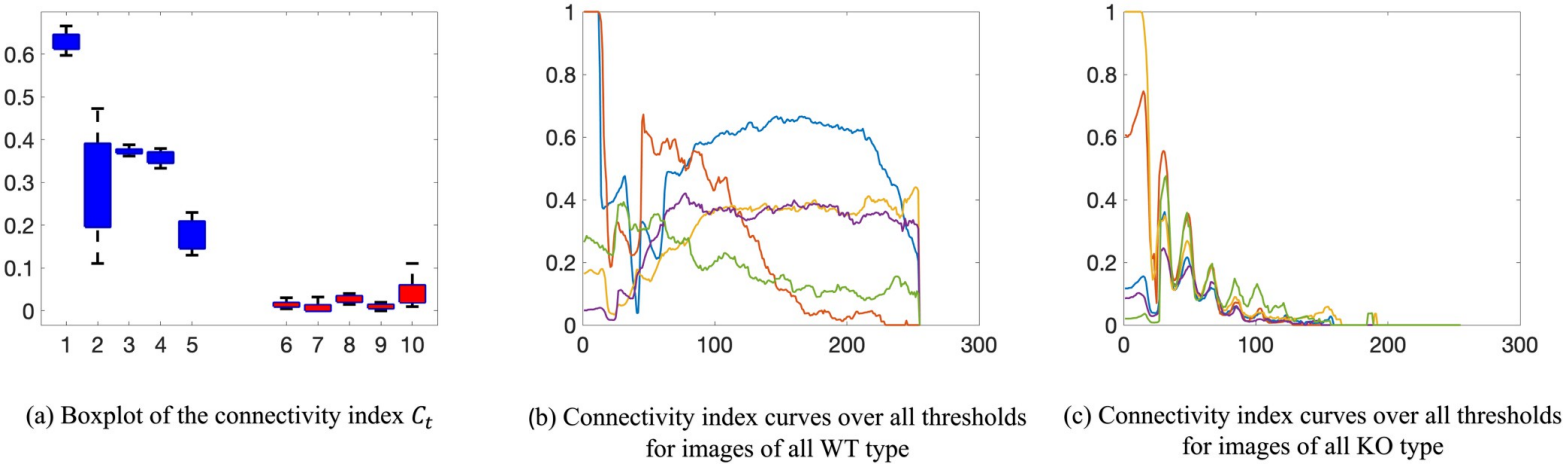
Comparison of connectivity index for WT and KO images. Connectivity index *C*_*t*_ is defined in (20), and the WT and KO images are shown in Fig 1. (a) Boxplot of the connectivity index *C*_*t*_ for *t* = 100, 101, ⋯, 150. The blue color indicates the WT type, and the red color indicates the KO type. Each number is a sample number and the dashed whiskers depict the range of the maximal and minimal values; (b) Connectivity index *C*_*t*_ for *t* = 0, 1, 2, 3, ⋯, 254, 255 for WT images; (c) Connectivity index *C*_*t*_ for *t* = 0, 1, 2, 3, ⋯, 254, 255 for KO images.

Fig 13(a) shows the boxplot of the connectivity index *C*_*t*_ for *t* = 100, 101, …, 150 for images of WT (blue) and KO (red) type. There are 5 different cell samples/images per genotype shown. We observe that the connectivity index of mitochondria WT cells is greater than the KO cells. This suggests that the WT mitochondria are more connected than the KO mitochondria. To further quantify their differences, we consider the connectivity index over all possible fluorescence signal threshold values as shown in Fig 13(b) and 13(c). We observe that the connectivity indices *C*_*t*_ for WT have more variability. Some cells are more connected than others. Such behavior was also observed in the case of the normalized Betti curve and the size distribution. On the other hand, the connectivity indices for KO cells have small variability because KO mitochondria networks in all the cell samples are quite fragmented.

### Statistical analysis

To investigate the feature behaviors of normalized Betti curve, size distribution of Betti numbers, and connectivity index on the sampled datasets shown in Figs 11–13, we performed statistical tests. Specifically, we consider the average values of the Normalized Betti curve and connectivity index curves over the threshold interval from 100 to 150 as the statistical targets for the hypothesis test (cf. Fig 13). On the other hand, based on the size distribution curves along with the sizes (the *x*-axis) of the structuring elements depicted in Fig 12, we consider the average values of the sampled size distribution curves over the size interval from 5 to 15. Using these average values as the target statistics in the hypothesis tests, permutation tests are performed and the results are depicted in Table 3. The permutation hypothesis tests are performed using the Python package mlxtend [72], with the significance level *α* set to 0.05. Demonstrations of the permutation hypothesis tests are also provided in our GitHub repository (see the “Code availability” section).

**Table 3:** Hypothesis tests of the proposed features for the sampled WT and KO mitochondria images.

Features	Feature range	*p*-value
$\rho_{1}^{H,0}\left(t\right)$	*t* ∈ [100, 150]	0.427 ± 0.017
$\rho_{1}^{H,3}\left(t\right)$	*t* ∈ [100, 150]	0.009 ± 0.003
$\rho_{1}^{H,5}\left(t\right)$	*t* ∈ [100, 150]	0.010 ± 0.003
*ϕ*(*D*_128_)(*s*)	*s* ∈ [5, 15]	0.025 ± 0.005
*C*_*t*_	*t* ∈ [100, 150]	0.009 ± 0.003

The proposed features are demonstrated in Figs 11–13. The statistics of the hypothesis tests are the average values of the WT and KO curves over the range [*a*, *b*], where the ranges are selected with reference to Figs 11–13. The test samples consist of 5 curves each from WT and KO mitochondria images. The null hypothesis, *H*_0_, states that the distribution of mean values of the WT and KO curves over the range [*a*, *b*] are the same. The permutation hypothesis test is performed by the python package mlxtend [72]. The *p*-value serves as the target value for determining the test results, and the decision value *α* is set to be 0.05. The hypotheses are repeated 100 times with different random seeds, and average values and standard deviations present the results. More detailed code for the statistical experiments can be found in our GitHub repository (see the “Code availability” section).

According to the results presented in Table 3, the proposed normalized Betti curves, size distribution of Betti numbers, and connectivity index methods demonstrate their potential in analyzing the differences and mutations between WT and KO mitochondrial structures. Based on the *p*-values from the experiments (0.009 ± 0.003, 0.010 ± 0.003, and 0.009 ± 0.003), curves $\rho_{1}^{H,3}$, $\rho_{1}^{H,5}$, and *C*_*t*_ achieve the most statistical significance for separating the WT and KO classes, showing that he WT and KO classes of images can be effectively separated based on these features. Furthermore, although the experiment of size distribution *ϕ*(*D*_128_)(*s*) yields a higher *p*-value and standard deviation (0.025 ± 0.005) compared to curves $\rho_{1}^{H,3}$, $\rho_{1}^{H,5}$, and *C*_*t*_, the result still rejects the null hypothesis that the WT and KO classes share the same mean of the size distributions in the range [5, 15]. Finally, we observe that the hypothesis test of the curve $\rho_{1}^{H,0}$ doesn’t show the statistical significance of the separation of WT and KO classes since the *p*-value (0.427 ± 0.017) is larger than the significance level *α* = 0.05. According to the definition (cf. (15)), the $\rho_{1}^{H,0}$ curves are produced by computing the conventional persistent homology based on the sub-level set filtration on a grayscale image. This statistical test demonstrates that, without morphological operations, the persistence information of images in the WT and KO classes is statistically indistinguishable. This finding indicates that the proposed morphology-based MF can provide more detailed geometric information for analyzing mitochondrial structures.

## Discussion and conclusion

In this work, we propose a novel mathematical framework combining persistent homology, mathematical morphology, and multiparameter filtration to study mitochondrial structural differences. The framework is flexible and can be applied to other types of cells. We also demonstrate ways to extract information from the bifiltration by developing three features for quantitating structural differences—normalized Betti curves, the size distribution of Betti numbers, and the connectivity index. They are independent of the size of each cell and robust to noise. Especially, with a main focus on demonstrating how the mathematical morphology operations integrate into the PH and MF framework, such as opening and thresholding operations, we consider the proposed three features that directly connect to the quantitative structural information within the mitochondria networks. In future investigations, a central focus will revolve around advancing an integrated machine learning model with the proficiency to autonomously determine parameters within the proposed features. This includes the adept selection of the sizes and shapes of the structuring elements. Concurrently, an additional avenue of exploration involves the integration of intrinsic features, notably the incorporation of persistence statistics [46], persistence images [47], and persistence landscapes [45]. This approach aims to delve deeper into the networks, providing a richer and more nuanced understanding.

Apart from the exploration of one-parameter persistent homology within the 2-dimensional persistence, a future focus of our study is the comprehensive investigation of 2-dimensional persistence. While the RIVET software [63] provides a framework for extracting persistence features and descriptors from a 2-dimensional bilfiltration, it is essential to note that the target filtration is founded on the point cloud within the $R^{n}$ space. This differs from the cubical complex filtration proposed in our work for the image [25]. Considering the 2-dimensional algebraic network induced by the bifiltration, a prospective avenue for future research involves a parallel implementation of RIVET’s feature extraction. This approach is crucial for aligning with our proposed image-based cubical complex filtration and holds the potential for enhancing the efficiency and relevance of our methodology. Furthermore, [1] proposed a *n*-parameter filtration on images via cubical complexes (where *n* > 2) based on alternating morphological operations. It would be interesting to utilize that *n*-parameter filtration to analyze the mitochondrial networks.

To the best of our knowledge, this work is one of the first ones that utilizes the one-persistent homology based on multiparameter filtrations to quantify mitochondrial networks. Recently, [12] used multiparameter persistence to study images of tumor cells. However, the methods in [12] of treating images and constructing multiparameter filtrations are fundamentally different than ours. In [12], point clouds were extracted from the grayscale images, and multiparameter filtration was constructed by distances and densities among those points. Our proposed method uses grayscale images and their smoothed images directly. There also have been studies that use one-parameter persistence to study other types of biological tissues, such as self-organization of biological tissues [73], fluorescence microscopy images [74], skin [75, 76], tumors [77], and other types of data [78].

There have been other mathematical models to analyze mitochondrial networks. For example, MiNA [79] is a widely used image macro tool to analyze mitochondrial networks and offers nine features to quantify mitochondrial networks. However, many of these approaches required manual thresholding. Different thresholds may result in different outcomes, and the choice of threshold is often subjective. We have addressed this limitation with our MF-PH analysis technique, which takes all threshold levels into consideration and is robust to noise. In particular, the proposed metrics—normalized Betti curves, the size distribution of Betti numbers, and the connectivity index—are scale-independent and dimensionless. Our approach is different than other groups who have developed automatic methods to segment mitochondria by a convolutional neural network [80–83].

From a clinical and scientific standpoint in bioimaging, obtaining microscopy images poses a significant challenge due to their scarcity and the complexities associated with extended experimental durations, labor-intensive processes, and resource-intensive requirements. Furthermore, images from distinct experiments often hinge on varied experimental assumptions and measurement outcomes, constraining the availability of consistent training samples and impeding the efficacy of machine learning mechanisms.

In light of these specific challenges, the employment of MF-PH methods emerges as a valuable approach. These methods offer a geometric and topological elucidation of mitochondrial networks, providing a more intuitive explanation and comprehensible understanding within the realm of medical theory. One limitation of the present study was that the proposed MF-PH framework was used on a limited sample size for each condition. Instead of cell lines, we utilized primary cells directly purified from our optineurin transgenic mice. Cells were purified from the embryos of transgenic mice. Due to the limited yield of transgenic knockout mice and the high cost of breeding these mice, our sample size was limited but sufficient to demonstrate the utility of our approach in this preliminary work. An anticipated future direction involves the utilization of our proposed MF-PH framework on an expanded set of microscopy images derived from diverse experimental conditions.

Our mathematical model and approach toward analyzing a complex biological structure using persistent homology will be useful in biomedical research and therapeutic development. In our paper, we have provided an example by applying this approach toward studying mitochondrial networks. Furthermore, we have created a “connectivity index” that summarizes all of these changes to allow quantitative comparison. Mitochondria structures take on complex shapes ranging from round or ovoid structures to long tubular networks. They can be individually isolated or connected through tubules in a branching pattern. This complex morphology presents a challenge for conventional image analysis methodology and software. However, using our approach, changes in the mitochondrial network in response to genetic mutations, aging, environmental insults, disease pathology, or drug treatment can be quantitated and compared. Beyond the mitochondrial network, further work will be required to apply our approach toward studying other complex biological structures such as endoplasmic reticulum, nuclear and cellular membranes, neuronal connectivity, and complex molecular structures.

## References

[1] Chung YM, Day S, Hu CS. A multi-parameter persistence framework for mathematical morphology. Scientific Reports. 2022;12(1):6427. doi: 10.1038/s41598-022-09464-7 35440703 PMC9019063

[2] Carlsson G, Vejdemo-Johansson M. Topological data analysis with applications. Cambridge University Press; 2021.

[3] Pritchard Y, Sharma A, Clarkin C, Ogden H, Mahajan S, Sánchez-García RJ. Persistent homology analysis distinguishes pathological bone microstructure in non-linear microscopy images. Scientific. 2023;13(1):2522. doi: 10.1038/s41598-023-28985-3 36781895 PMC9925777

[4] Aukerman A, Carrière M, Chen C, Gardner K, Rabadán R, Vanguri R. Persistent homology based characterization of the breast cancer immune microenvironment: a feasibility study. Journal of Computational Geometry. 2022;12(2):183–206.

[5] Hu X, Li F, Samaras D, Chen C. Topology-preserving deep image segmentation. Advances in neural information processing systems. 2019;32. PMC11526043 39484069

[6] Gupta S, Hu X, Kaan J, Jin M, Mpoy M, Chung K, et al. Learning topological interactions for multi-class medical image segmentation. In: European Conference on Computer Vision. Springer; 2022. p. 701–718.

[7] Carlsson G, Singh G, Zomorodian AJ. Computing multidimensional persistence. Journal of Computational Geometry. 2010;1(1):72–100.

[8] Botnan MB, Lesnick M. An introduction to multiparameter persistence; 2022.

[9] Xia K, Wei GW. Multidimensional persistence in biomolecular data. Journal of Computational Chemistry, 36, 1502–1520; 2015. doi: 10.1002/jcc.23953 26032339 PMC4485576

[10] Chen Y, Segovia-Dominguez I, Akcora CG, Zhen Z, Kantarcioglu M, Gel Y, et al. EMP: Effective Multidimensional Persistence for Graph Representation Learning. In: Learning on Graphs Conference. PMLR; 2024. p. 24–1.

[11] Demir A, Coskunuzer B, Gel Y, Segovia-Dominguez I, Chen Y, Kiziltan B. ToDD: Topological compound fingerprinting in computer-aided drug discovery. Advances in Neural Information Processing Systems. 2022;35:27978–27993.

[12] Vipond O, Bull JA, Macklin PS, Tillmann U, Pugh CW, Byrne HM, et al. Multiparameter persistent homology landscapes identify immune cell spatial patterns in tumors. Proceedings of the National Academy of Sciences. 2021;118(41):e2102166118. doi: 10.1073/pnas.2102166118 34625491 PMC8522280

[13] Albagha OM, Visconti MR, Alonso N, Langston AL, Cundy T, Dargie R, et al. Genome-wide association study identifies variants at CSF1, OPTN and TNFRSF11A as genetic risk factors for Paget’s disease of bone. Nature genetics. 2010;42(6):520–524. doi: 10.1038/ng.562 20436471 PMC3217192

[14] Cirulli ET, Lasseigne BN, Petrovski S, Sapp PC, Dion PA, Leblond CS, et al. Exome sequencing in amyotrophic lateral sclerosis identifies risk genes and pathways. Science. 2015;347(6229):1436–1441. doi: 10.1126/science.aaa3650 25700176 PMC4437632

[15] Aung T, Rezaie T, Okada K, Viswanathan AC, Child AH, Brice G, et al. Clinical features and course of patients with glaucoma with the E50K mutation in the optineurin gene. Investigative ophthalmology & visual science. 2005;46(8):2816–2822. doi: 10.1167/iovs.04-1133 16043855

[16] Rezaie T, Child A, Hitchings R, Brice G, Miller L, Coca-Prados M, et al. Adult-onset primary open-angle glaucoma caused by mutations in optineurin. Science. 2002;295(5557):1077–1079. doi: 10.1126/science.1066901 11834836

[17] Evans CS, Holzbaur EL. Degradation of engulfed mitochondria is rate-limiting in Optineurin-mediated mitophagy in neurons. Elife. 2020;9:e50260. doi: 10.7554/eLife.50260 31934852 PMC6959996

[18] Wong YC, Holzbaur EL. Optineurin is an autophagy receptor for damaged mitochondria in parkin-mediated mitophagy that is disrupted by an ALS-linked mutation. Proceedings of the National Academy of Sciences. 2014;111(42):E4439–E4448. doi: 10.1073/pnas.1405752111 25294927 PMC4210283

[19] Lazarou M, Sliter DA, Kane LA, Sarraf SA, Wang C, Burman JL, et al. The ubiquitin kinase PINK1 recruits autophagy receptors to induce mitophagy. Nature. 2015;524(7565):309–314. doi: 10.1038/nature14893 26266977 PMC5018156

[20] Wong SW, Huang BW, Hu X, Ho Kim E, Kolb JP, Padilla RJ, et al. Global deletion of Optineurin results in altered type I IFN signaling and abnormal bone remodeling in a model of Paget’s disease. Cell Death & Differentiation. 2020;27(1):71–84. doi: 10.1038/s41418-019-0341-6 31076632 PMC7205997

[21] Tseng HC, Riday TT, McKee C, Braine CE, Bomze H, Barak I, et al. Visual impairment in an optineurin mouse model of primary open-angle glaucoma. Neurobiology of aging. 2015;36(6):2201–2212. doi: 10.1016/j.neurobiolaging.2015.02.012 25818176 PMC4433607

[22] Carlsson G, Singh G, Zomorodian A. Computing Multidimensional Persistence. In: Algorithms and Computation. Berlin, Heidelberg: Springer Berlin Heidelberg; 2009. p. 730–739.

[23] Scaramuccia S, Iuricich F, De Floriani L, Landi C. Computing multiparameter persistent homology through a discrete Morse-based approach. Computational Geometry. 2020;89:101623. doi: 10.1016/j.comgeo.2020.101623

[24] Harrington HA, Otter N, Schenck H, Tillmann U. Stratifying multiparameter persistent homology. SIAM Journal on Applied Algebra and Geometry. 2019;3(3):439–471. doi: 10.1137/18M1224350

[25] Kaczynski T, Mischaikow K, Mrozek M. Computational Homology. Applied Mathematical Sciences. New York, Inc.: Springer-Verlag; 2006.

[26] Gonzalez RC, Woods RE. Digital Image Processing. Digital Image Processing. Prentice Hall; 2002.

[27] Sonka M, Hlavac V, Boyle R. Image Processing, Analysis, and Machine Vision. Cengage Learning; 2014.

[28] Sezgin M, Sankur B. Survey over image thresholding techniques and quantitative performance evaluation. Journal of Electronic imaging. 2004;13(1):146–165. doi: 10.1117/1.1631315

[29] Otsu N. A threshold selection method from gray-level histograms. IEEE transactions on systems, man, and cybernetics. 1979;9(1):62–66. doi: 10.1109/TSMC.1979.4310076

[30] Chung YM, Day S. Topological fidelity and image thresholding: A persistent homology approach. Journal of Mathematical Imaging and Vision. 2018;60(7):1167–1179. doi: 10.1007/s10851-018-0802-4

[31] Serra J. Image Analysis and Mathematical Morphology. No. 1 in Image Analysis and Mathematical Morphology. Academic Press; 1984.

[32] Soille P. Morphological Image Analysis: Principles and Applications. 2nd ed. Secaucus, NJ, USA: Springer-Verlag New York, Inc.; 2003.

[33] Najman L, Talbot H. Mathematical Morphology. 1st ed. Wiley-ISTE; 2010.

[34] Haralick RM, Sternberg SR, Zhuang X. Image Analysis Using Mathematical Morphology. IEEE Transactions on Pattern Analysis and Machine Intelligence. 1987;PAMI-9(4):532–550. doi: 10.1109/TPAMI.1987.4767941 21869411

[35] Ritter GX, Sussner P. An introduction to morphological neural networks. In: Proceedings of 13th International Conference on Pattern Recognition. vol. 4; 1996. p. 709–717 vol.4.

[36] Dougherty ER, Sinha D. Computational mathematical morphology. Signal Processing. 1994;38(1):21–29. doi: 10.1016/0165-1684(94)90054-X

[37] Goy A, Aiguier M, Bloch I. From Structuring Elements to Structuring Neighborhood Systems. In: Burgeth B, Kleefeld A, Naegel B, Passat N, Perret B, editors. Mathematical Morphology and Its Applications to Signal and Image Processing. Cham: Springer International Publishing; 2019. p. 16–28.

[38] Cousty J. Segmentation, hierarchy, mathematical morphology filtering, and application to image analysis [Habilitation à diriger des recherches]. Université Paris-Est; 2018.

[39] Greenberg MJ, Harper JR. Algebraic Topology, A First Course. Addison-Wesley Publishing Company; 1980.

[40] Vick JW. Homology Theory, A Introduction to Algebraic Topology. Springer-Verlag Publishing Company, Second Edition; 1973.

[41] Munkres JR. Elements Of Algebraic Topology. CRC Press; 2018.

[42] Hatcher A. Algebraic topology. Cambridge: Cambridge Univ. Press; 2000.

[43] Chen L, Rong Y. Digital topological method for computing genus and the Betti numbers. Topology and its Applications. 2010;157(12):1931–1936. doi: 10.1016/j.topol.2010.04.006

[44] Sossa-Azuela JH, Santiago-Montero R, Pérez-Cisneros M, Rubio-Espino E. Computing the Euler Number of a Binary Image Based on a Vertex Codification. Journal of Applied Research and Technology. 2013;11(3):360–370. doi: 10.1016/S1665-6423(13)71546-3

[45] Bubenik P. Statistical Topological Data Analysis using Persistence Landscapes. Journal of Machine Learning Research. 2015;16(3):77–102.

[46] Bubenik P, Dłotko P. A persistence landscapes toolbox for topological statistics. Journal of Symbolic Computation. 2017;78:91–114. doi: 10.1016/j.jsc.2016.03.009

[47] Adams H, Emerson T, Kirby M, Neville R, Peterson C, Shipman P, et al. Persistence Images: A Stable Vector Representation of Persistent Homology. Journal of Machine Learning Research. 2017;18(8):1–35.

[48] Panconi L, Makarova M, Lambert ER, May RC, Owen DM. Topology-based fluorescence image analysis for automated cell identification and segmentation. Journal of Biophotonics. 2023;16(3):e202200199. doi: 10.1002/jbio.202200199 36349740

[49] Edelsbrunner H, Letscher D, Zomorodian A. Topological Persistence and Simplification. Discrete Comput Geom. 2002;28:511–533. doi: 10.1007/s00454-002-2885-2

[50] Carlsson G, Zomorodian A, Collins A, Guibas L. Persistence Barcodes for Shapes. International Journal of Shape Modeling. 2005;11:149–188. doi: 10.1142/S0218654305000761

[51] Zomorodian A, Garlsson G. Computing Persistent Homology. ACM, Discrete and Computational Geometry, Volume 33 Issue 2, February 2005 Pages 249–274; 2005. doi: 10.1007/s00454-004-1146-y

[52] Carlsson G. Topology and Data. Bull. Amer. Math. Soc. 46 (2009), 255–308; 2009. doi: 10.1090/S0273-0979-09-01249-X

[53] Edelsbrunner H, Harer J. Computational Topology: An Introduction. American Mathematical Society; 2010.

[54] Edelsbrunner H. Persistent homology: theory and practice. Bulletin of the American Mathematical Society. 2014;.

[55] Hu CS, Chung YM. On the Conditions of Absorption Property for Morphological Opening and Closing; 2020.

[56] Otter N, Porter MA, Tillmann U, Grindrod P, Harrington HA. A roadmap for the computation of persistent homology. EPJ Data Science. 2017;6:1–38. doi: 10.1140/epjds/s13688-017-0109-5 32025466 PMC6979512

[57] Nanda V. Perseus, the Persistent Homology Software.; 2013. http://www.sas.upenn.edu/~vnanda/perseus.

[58] Mischaikow K, Nanda V. Morse theory for filtrations and efficient computation of persistent homology. Discrete & Computational Geometry. 2013;50:330–353. doi: 10.1007/s00454-013-9529-6

[59] Kashiwara M, Schapira P. Piecewise Linear Sheaves. International Mathematics Research Notices. 2019. doi: 10.1093/imrn/rnz145

[60] Kashiwara M, Schapira P. Persistent homology and microlocal sheaf theory. Journal of Applied and Computational Topology. 2018;2. doi: 10.1007/s41468-018-0019-z

[61] Loiseaux D, Carrière M, Blumberg A. A framework for fast and stable representations of multiparameter persistent homology decompositions. Advances in Neural Information Processing Systems. 2024;36.

[62] Botnan M, Crawley-Boevey W. Decomposition of persistence modules. Proceedings of the American Mathematical Society. 2020;148(11):4581–4596. doi: 10.1090/proc/14790

[63] The RIVET Developers. RIVET; 2020. Available from: https://github.com/rivetTDA/rivet/.

[64] Richardson E, Werman M. Efficient classification using the Euler characteristic. Pattern Recognition Letters. 2014;49:99–106. doi: 10.1016/j.patrec.2014.07.001

[65] Chung YM, Lawson A. Persistence curves: A canonical framework for summarizing persistence diagrams. Advances in Computational Mathematics. 2022;48(1):6. doi: 10.1007/s10444-021-09893-4

[66] Heiss T, Wagner H. Streaming Algorithm for Euler Characteristic Curves of Multidimensional Images. In: Felsberg M, Heyden A, Krüger N, editors. Computer Analysis of Images and Patterns. Cham: Springer International Publishing; 2017. p. 397–409.

[67] Fasy BT, Micka S, Millman DL, Schenfisch A, Williams L. Challenges in Reconstructing Shapes from Euler Characteristic Curves; 2018.

[68] Klein JC, Serra J. The texture analyser. Journal of Microscopy. 1972;95(2):349–356. doi: 10.1111/j.1365-2818.1972.tb03734.x

[69] Colomer A, Igual J, Naranjo V. Detection of Early Signs of Diabetic Retinopathy Based on Textural and Morphological Information in Fundus Images. Sensors. 2020;20(4):1005. doi: 10.3390/s20041005 32069912 PMC7071097

[70] Gager V, Legland D, Bourmaud A, Le Duigou A, Pierre F, Behlouli K, et al. Oriented granulometry to quantify fibre orientation distributions in synthetic and plant fibre composite preforms. Industrial Crops and Products. 2020;152:112548. doi: 10.1016/j.indcrop.2020.112548

[71] The GUDHI Project. GUDHI User and Reference Manual. 3.1.1 ed. GUDHI Editorial Board; 2020. Available from: https://gudhi.inria.fr/doc/3.1.1/.

[72] Raschka S. MLxtend: Providing machine learning and data science utilities and extensions to Python’s scientific computing stack. The Journal of Open Source Software. 2018;3(24). doi: 10.21105/joss.00638

[73] Jimenez MJ, Rucco M, Vicente-Munuera P, Gómez-Gálvez P, Escudero LM. Topological data analysis for self-organization of biological tissues. In: International Workshop on Combinatorial Image Analysis. Springer; 2017. p. 229–242.

[74] Edwards P, Skruber K, Milićević N, Heidings JB, Read TA, Bubenik P, et al. TDAExplore: Quantitative analysis of fluorescence microscopy images through topology-based machine learning. Patterns. 2021;2(11):100367. doi: 10.1016/j.patter.2021.100367 34820649 PMC8600226

[75] Hu CS, Lawson A, Chen JS, Chung YM, Smyth C, Yang SM. Toporesnet: A hybrid deep learning architecture and its application to skin lesion classification. Mathematics. 2021;9(22):2924. doi: 10.3390/math9222924

[76] Koseki K, Kawasaki H, Atsugi T, Nakanishi M, Mizuno M, Naru E, et al. Assessment of skin barrier function using skin images with topological data analysis. NPJ systems biology and applications. 2020;6(1):1–9. doi: 10.1038/s41540-020-00160-8 33339832 PMC7749164

[77] Lawson P, Sholl AB, Brown JQ, Fasy BT, Wenk C. Persistent homology for the quantitative evaluation of architectural features in prostate cancer histology. Scientific Reports. 2019;9(1):1–15. doi: 10.1038/s41598-018-36798-y 30718811 PMC6361896

[78] Amézquita EJ, Quigley MY, Ophelders T, Munch E, Chitwood DH. The shape of things to come: Topological data analysis and biology, from molecules to organisms. Developmental Dynamics. 2020;249(7):816–833. doi: 10.1002/dvdy.175 32246730 PMC7383827

[79] Valente AJ, Maddalena LA, Robb EL, Moradi F, Stuart JA. A simple ImageJ macro tool for analyzing mitochondrial network morphology in mammalian cell culture. Acta histochemica. 2017;119(3):315–326. doi: 10.1016/j.acthis.2017.03.001 28314612

[80] Xiao C, Chen X, Li W, Li L, Wang L, Xie Q, et al. Automatic Mitochondria Segmentation for EM Data Using a 3D Supervised Convolutional Network. Frontiers in Neuroanatomy. 2018;12:92. doi: 10.3389/fnana.2018.00092 30450040 PMC6224513

[81] Leonard AP, Cameron RB, Speiser JL, Wolf BJ, Peterson YK, Schnellmann RG, et al. Quantitative analysis of mitochondrial morphology and membrane potential in living cells using high-content imaging, machine learning, and morphological binning. Biochimica et Biophysica Acta (BBA)—Molecular Cell Research. 2015;1853(2):348–360. doi: 10.1016/j.bbamcr.2014.11.002 25447550 PMC4289477

[82] Zahedi A, On V, Phandthong R, Chaili A, Remark G, Bhanu B, et al. Deep Analysis of Mitochondria and Cell Health Using Machine Learning. Scientific Reports. 2018;. doi: 10.1038/s41598-018-34455-y 30397207 PMC6218515

[83] Song OR, Brodin P, Buchrieser C, Escoll P. Mitochondrial dynamics and activity in legionella-infected cells. Legionella: Methods and Protocols. 2019; p. 205–220. doi: 10.1007/978-1-4939-9048-1_13 30694494

