# Correction: Morphological multiparameter filtration and persistent homology in mitochondrial image analysis

**DOI:** 10.1371/journal.pone.0330651

**Published:** 2025-08-20

**Authors:** Yu-Min Chung, Chuan-Shen Hu, Emily Sun, Henry C. Tseng

The corresponding author’s email address is incorrect. Chuan-Shen Hu’s email address is: chuanshenhu.official@gmail.com
